# HMOX1 promotes lung adenocarcinoma metastasis by affecting macrophages and mitochondrion complexes

**DOI:** 10.3389/fonc.2022.978006

**Published:** 2022-08-12

**Authors:** Bo Chen, Liyang Zhang, Hongshu Zhou, Wenrui Ye, Cong Luo, Liting Yang, Ning Fang, Anliu Tang

**Affiliations:** ^1^ Department of Neurosurgery, Xiangya Hospital, Central South University, Changsha, China; ^2^ National Clinical Research Center for Geriatric Disorders, Xiangya Hospital, Central South University, Changsha, China; ^3^ Department of Urology, Xiangya Hospital, Central South University, Changsha, China; ^4^ Department of Gastroenterology, The Third Xiangya Hospital of Central South University, Changsha, China; ^5^ Hunan Key Laboratory of Nonresolving Inflammation and Cancer, Central South University, Changsha, China

**Keywords:** HMOX1, lung adenocarcinoma, metastasis, prognostic model, drug prediction

## Abstract

**Background:**

Metastasis is the leading cause of lung adenocarcinoma (LUAD) patient death. However, the mechanism of metastasis is unclear. We performed bioinformatic analyses for HMOX1 (Heme oxygenase-1), aiming to explore its role in LUAD metastasis.

**Methods:**

Pan-cancer analysis was first used to identify the metastasis-associated role of HMOX1 in LUAD. HMOX1-related genomic alterations were then investigated. Based on functional enrichment, we systematically correlated HMOX1 with immunological characteristics and mitochondrial activities. Furthermore, weighted gene co-expression network analysis (WGCNA) was applied to construct the HMOX1-mediated metastasis regulatory network, which was then validated at the proteomic level. Finally, we conducted the survival analysis and predicted the potential drugs to target the HMOX1 network.

**Results:**

HMOX1 expression was significantly associated with epithelial-mesenchymal transition (EMT) and lymph and distant metastasis in LUAD. High HMOX1 levels exhibited higher macrophage infiltration and lower mitochondrial complex expression. WGCNA showed a group of module genes co-regulating the traits mentioned above. Subsequently, we constructed an HMOX1-mediated macrophage-mitochondrion-EMT metastasis regulatory network in LUAD. The network had a high inner correlation at the proteomic level and efficiently predicted prognosis. Finally, we predicted 9 potential drugs targeting HMOX1-mediated metastasis in LUAD, like chloroxine and isoliquiritigenin.

**Conclusions:**

Our analysis elaborates on the role of HMOX1 in LUAD metastasis and identified a highly prognostic HMOX1-mediated metastasis regulatory network. Novel potential drugs targeting the HMOX1 network were also proposed, which should be tested for their activity against LUAD metastasis in future studies.

## Introduction

Lung cancer is histologically divided into small cell lung cancer (SCLC, 15% patients) and non-small cell lung cancer (NSCLC, 85% of patients). NSCLC has been classified into three types: adenocarcinoma, squamous cell carcinoma, and large cell. Lung adenocarcinoma (LUAD) is the most common subtype of NSCLC and represents nearly 40% of all lung cancers (1). Frustratingly, only 26% of LUAD and squamous cell carcinoma patients survive 5 years after diagnosis (2). LUAD is often detected at the metastatic stage with prevalence in bone and nervous system (3), and metastasis is the cause of most LUAD patient deaths. Even with therapy, the 5-year relative survival rate for patients suffering metastasis is approximately 6% (2). Unfortunately, the mechanism of LUAD metastasis remains largely unknown, limiting the development of therapeutic drugs.

The process of cancer metastasis mainly consists of 5 essential steps: invasion, intravasation, circulation, extravasation, and colonization (4). At the metastasis initiation stage, epithelial-mesenchymal transition (EMT), a dynamic process where cells abandon their epithelial features and transform into a more mesenchymal phenotype, exerts a vital role (5). EMT is considered as a common characteristic for disseminated tumor cells and circulating tumor cells (6), and EMT cells modulate immune system processes. Cells undergoing EMT can alter the tumor immune microenvironment, which in further induces the EMT process. Many immune cells, like macrophages, neutrophils, T cells, and others, have the potential to promote EMT and further accelerate tumor metastasis (7). In addition, EMT requires a huge energy supply from mitochondria, and inhibition of the mitochondrial complex can hinder tumor growth and metastasis (8).

Heme oxygenase-1 (HMOX1) is an inducible intracellular enzyme to degrade heme, expressed in both malignant tumor cells and tumor-associated macrophages (TAMs) (9). At the level of organelles, HMOX1 mainly localizes to the endoplasmic reticulum, mitochondrion, and others (10, 11). Intrinsically, HMOX1 activity provides cytoprotective effects, including anti-oxidation, anti-apoptosis, and heme clearance. However, HMOX1 has also been demonstrated to promote tumor progression and metastasis in multiple cancers such as glioma, colorectal cancer, melanoma, breast cancer, and others (9). In addition, HMOX1 is functionally associated with TAM polarization and mitochondrial dysfunction (12, 13). Currently, pharmacological inhibition of HMOX1 is considered a promising therapeutic approach to hinder tumor metastasis. However, the role of HMOX1 in metastasis still remains unclear and needs to be fully elucidated (9).

This study aimed to elaborate on the role of HMOX1 in LUAD metastasis through multi-omics analyses including genomics, transcriptomics, and proteomics. The association between HMOX1 and LUAD metastasis was first identified by its relationship to EMT, lymph, and distant metastasis. Next, we investigated associations with the immune microenvironment and mitochondrial pathways and constructed an HMOX1-mediated macrophage-mitochondrion-EMT metastasis regulatory network by weighted gene co-expression network analysis (WGCNA). A prognostic model of the regulatory network was then developed. Potential drugs, like chloroxine and isoliquiritigenin, were also predicted to inhibit the HMOX1-mediated LUAD metastasis.

## Methods

**Supplemental Figure 1** shows the workflow of this research.

### Data collection and preprocessing

The transcriptome data: the Cancer Genome Atlas (TCGA) pan-cancer data and 4 Gene Expression Omnibus (GEO) LUAD cohorts (GSE11969, GSE31210, GSE42127, GSE68465) with their clinical information were separately downloaded from the UCSC Xena and the GEO data portals (https://portal.gdc.cancer.gov/, https://www.ncbi.nlm.nih.gov/geo/). The FPKM value of TCGA sequencing data was transformed into the TPM value. GEO microarray data had 3 different platforms: Illumina, Agilent, and Affymetrix. The normalized microarray data from Illumina maintained the original format. The raw microarray data from Agilent and Affymetrix were processed using the “limma” and “oligo” packages, separately. In addition, the LUAD genome data, including somatic copy number alternations (CNAs) and mutations, which corresponded to the cases with the transcriptome data, were downloaded from the TCGA database. The proteomic data with paired mRNA datasets were obtained from the National Cancer Institute’s Clinical Proteomic Tumor Analysis Consortium (CPTAC) portal (https://proteomics.cancer.gov/data-portal). The detailed information of these datasets was listed in **Table 1**.

**Table 1:** The datasets used in this study.

Data source	Omics category	Platform	Sample count
TCGA (pan-cancer)	mRNA	Illumina HiSeq 2000	10277
TCGA (LUAD)	mRNA	Illumina HiSeq 2000	515
TCGA (LUAD)	genome	Affymetrix SNP 6.0	516
GSE11969	mRNA	Agilent Homo 2.16 K	90
GSE31210	mRNA	Affymetrix Plus 2.0	226
GSE42127	mRNA	Illumina WG-6 V3.0	133
GSE68465	mRNA	Affymetrix U133A	441
CPTAC	mRNA	Illumina Hiseq 4000	111
CPTAC	protein	Tandem mass tags	111

### Tumor metastasis

Tumor metastasis potential was evaluated by analysis of EMT, lymph metastasis, and distant metastasis. We collected 77 EMT signatures from a previous study (14). Mesenchymal and epithelial phenotypes were identified by signature gene expression and quantified by Gene Set Variation Analysis scores (GSVA) (15). Furthermore, we identified the lymph and distant metastasis according to pathological TMN staging (N0: no lymph metastasis, N1 – 3: lymph metastasis; M0: no distant metastasis; M1: distant metastasis). To confirm the role of HMOX1 in modulating tumor metastasis, we analyzed the correlation between HMOX1 and metastasis characteristics mentioned above in the terms of pan-cancer and LUAD. In addition, we investigated the HMOX1 expression disparity in primary tumors and multiple metastasis sites in the Human Cancer Metastasis Database (HCMDB, http://hcmdb.i-sanger.com/index).

### Genomic alteration

We used cBioPortal (http://www.cbioportal.org/) to identify the CNA and mutation frequency of HOMX1 in the LUAD. Subsequently, we compared the genomic features of LUAD patients with different HMOX1 expression levels in the TCGA database. Arm- and focal-level somatic CNAs were calculated using GISTIC 2.0 (https://cloud.genepattern.org/). Significantly mutated genes and their interaction effect were identified using maftools.

### Functional annotation

To explore other HMOX1-related functional phenotypes, we performed functional annotation for HMOX1. The 33.3th and 66.6th percentiles of HMOX1 expression were set as the cutoffs to divide LUAD patients into low, medium, and high groups. The differentially expressed genes (DEGs) between the low and high groups were screened using the “limma” package (P < 0.05). Subsequently, the biological functions of DEGs were annotated by Gene Ontology (GO) and Kyoto Encyclopedia of Genes and Genomes (KEGG).

### Immune infiltration

Immune cells can contribute to tumor EMT, invasion, and metastasis. We first investigated 7 cancer immunity cycles in LUAD as previously reported (16). Next, the immunocyte infiltration was estimated at the Cancer Immunome Atlas (TCIA, https://tcia.at/home) and TIMER2.0 (http://timer.cistrome.org/). To reduce the estimation mistakes, we comprehensively applied 9 different algorithms: GSEA (NES>0, FDR<0.1; TCIA website), CIBERSORT, CIBERSORT-ABS, QUANRISEQ, XCELL, TIDE, EPIC, TIMER, MCPCOUNTER (TIMER2.0 website). In addition, we also investigated the expression of immunocyte effector genes and 22 immune checkpoint markers according to the previous work (16). To explore the role of HMOX1 in LUAD immune regulation, we performed the correlation analysis between the HMOX1 and immune characteristics with respect to the above.

### Mitochondrion

Mitochondria play a crucial role in tumor cell migration, invasion, and metastasis (17). We downloaded 41 mitochondria-related pathways from the MITOCARTA3.0 database (https://www.broadinstitute.org/mitocarta/mitocarta30-inventory-mammalian-mitochondrial-proteins-and-pathways). The enrichment scores of these pathways were calculated using the “GSVA” package (15). Subsequently, we further analyzed the expression of 5 mitochondrial complexes in LUAD by feature genes and pathway enrichment.

### Co-regulatory network construction

To explore the correlation between HMOX1-mediated metastasis and other functional phenotypes, we performed weighted gene co-expression network analysis using the “WGCNA” package. A power of β = 6 and a scale-free R^2^ = 0.98 was set to attain a scale-free topology network. We extracted the closest-associated module with HMOX1 expression, EMT traits, macrophage infiltration, and mitochondrial complex level, which was assumed to be the key module involved in LUAD metastasis. The gene function of the key module was analyzed by GO enrichment.

Next, we intersected the module genes in the key module and phenotype feature genes. The signature genes of EMT and mitochondrial complex have been mentioned above. The macrophage signature genes were derived from the top 5 macrophage terms in the GO enrichment. The intersected genes were then imported into the STRING database with the minimum required interaction score of 0.4 (https://www.string-db.org/). Protein-protein network was further visualized by Cytoscape software (version 3.9.0).

### Proteomic analysis

For proteomics, we first performed a differential protein expression analysis between the high and low HMOX1 groups (the student’s t-test, *P* < 0.05). GO functional enrichment was then conducted. In terms of the paired sample mRNAs, we estimated the expression level of EMT, mitochondrial complex, and macrophage using the aforementioned methods. After that, we applied the disparity comparison and correlation analysis to the phenotype scores and feature gene expression, to explore the inner correlation of the HMOX1-mediated macrophage-mitochondrion-EMT network at the protein level.

### Survival analysis

The TCGA-LUAD cohort was randomly divided into the training set and internal validation set, with a ratio of 7:3. The 4 GEO cohorts were selected as external validation sets. In the training set, we conducted the univariate Cox regression in the HMOX1-mediated metastasis regulatory network and its neighbor genes. Next, the least absolute shrinkage and selection operator (Lasso) algorithm was used to screen genes with optimal prognosis prediction ability. We then developed risk scores using these genes as follows:Σ regression coefficient * gene expression. The optimal cut-off was ascertained to divide the high and low groups by the surv_cutpoint function of the “survminer” packages. Kaplan-Meier curves with the log-rank test were then used to compare survival rates between the two groups. The predictive ability of risk scores was also assessed by the AUC values.

Furthermore, univariate and multivariate Cox regressions were applied to assess the independent prognostic ability of the risk score and other clinical variables. A nomogram consisting of independent prognostic factors was developed using the “rms” package and internally and externally validated by calibration and ROC curves.

### Small molecular drug analysis

The Connectivity Map (CMAP) website (https://clue.io/) was applied to explore small molecule drugs with the potential to inhibit HMOX1-mediated LUAD metastasis. The genes in the co-regulatory network were uploaded. The drugs with negative Raw_cs and high fdr_q_nlog10 values were considered as potential therapeutic agents because they could suppress the expression of these genes.

### Statistical analysis

For continuous variables, the Wilcoxon test was used to examine the difference between binary groups while the Kruskal-Wallis test was employed for multiple groups. Categorical variables were compared using Fisher’s exact test. In addition, the correlation between variables was examined by the Spearman coefficient. All statistical tests were two-sided and *P* < 0.05 was considered statistically significant. Statistical analysis and data visualization was conducted using R version 4.1.2, Matlab version 2021, and Sangerbox version 3.0 (http://vip.sangerbox.com/home.html).

## Results

### HMOX1 is correlated to metastasis across cancer types and in LUAD

**Figure 1:**
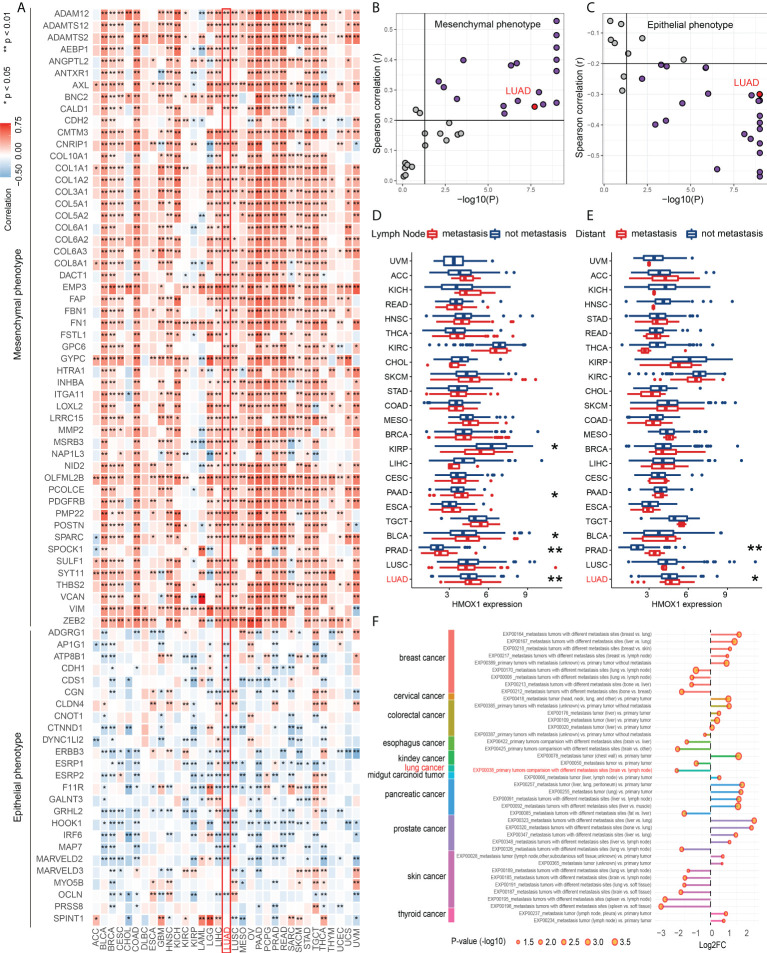
The effect of HMOX1 on metastasis across cancer types. **(A)** Correlation between HMOX1 and 77 epithelium-mesenchymal transition (EMT) signature genes. **(B)** Correlation between HMOX1 and mesenchymal phenotype scoring. **(C)** Correlation between HMOX1 and epithelial phenotype scoring. **(D)** Difference in HMOX1 expression between lymph node metastasis and without metastasis groups. **(E)** Difference in HMOX1 expression between distant metastasis and without metastasis groups **(F)** Multiple types of metastasis comparisons for the HMOX1 expression in the HCMDB database. *P < 0.05; **P < 0.01.

Pan-cancer analyses were performed to investigate the role of HMOX1 in metastasis regulation. Our findings showed that HMOX1 was positively associated with a mesenchymal-like phenotype and was negatively associated with an epithelial phenotype in most tumors, including LUAD (**Figures 1A–C**). In addition, tumors with lymph node and/or distant metastasis had significantly higher HMOX1 expression than tumors without metastasis, including prostate adenocarcinoma (PRAD) and LUAD (**Figures 1D, E**). HCMDB data unveiled that HMOX1 was extensively differentially expressed in the multiple types of metastasis comparisons (primary tumors with metastasis *vs*. primary tumors without metastasis; primary tumors *vs*. metastatic tumors; metastatic tumors with different metastasis sites) in the various tumors, including LUAD (**Figure 1F**).

**Figure 2:**
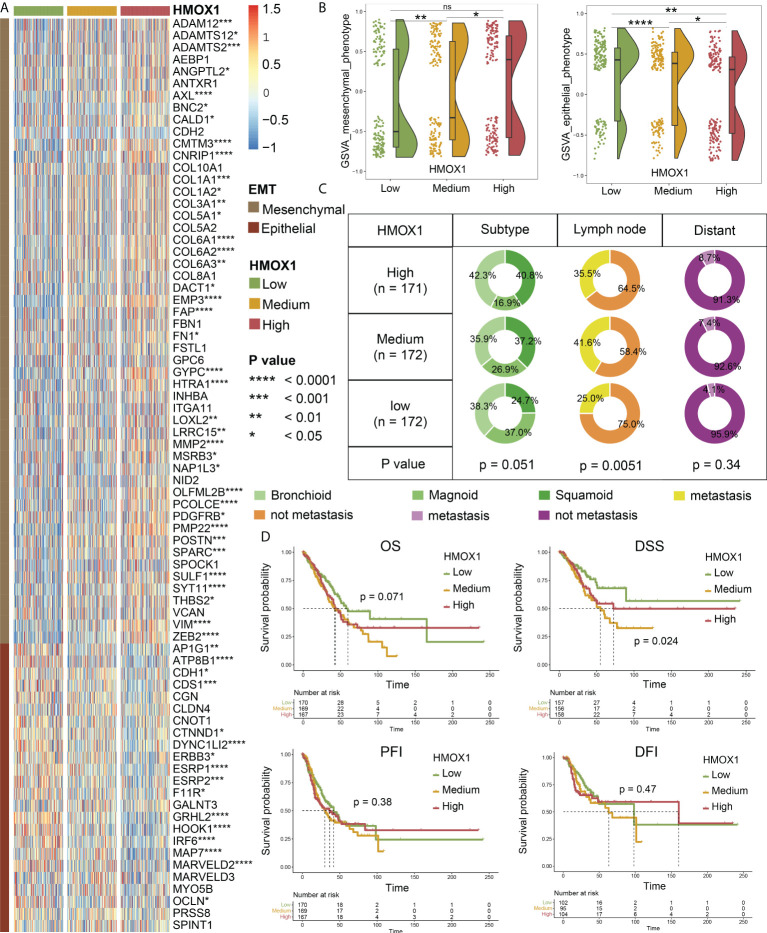
The effect of HMOX1 on metastasis in lung adenocarcinoma (LUAD). **(A)** Differences in the expression of EMT signature genes among high, medium, and low HMOX1 groups. **(B)** Differences in the EMT scoring between high, medium, and low HMOX1 groups. **(C)** Differences in the LUAD subtype, lymph node metastasis, and distant metastasis among high, medium, and low HMOX1 groups. **(D)** Survival analyses for the high, medium, and low HMOX1 groups. OS, overall survival; DSS, disease-specific survival; PFI, progress-free interval; DFI, disease-free interval. NS, not significant; *P < 0.05; ** P< 0.01; ***P < 0.001; ****P < 0.0001.

Next, we focused on LUAD and divided the patients into low, medium, and high groups according to the HMOX1 expression. Heatmap and violin plots showed that the high HMOX1 group had higher expression of the mesenchymal phenotype, followed by the medium and low groups. The opposite trend was observed for the epithelial phenotype (**Figures 2A, B**). Furthermore, we compared the clinical traits and found that the proportions of patients with squamoid subtype, lymph node metastasis, and distant metastasis in the high and medium HMOX1 groups were higher than those in the low group (**Figure 2C**). Survival analysis suggested that low HMOX1 samples showed a better disease-specific survival (DSS) prognosis than medium and high samples in LUAD. However, we failed to observe the statistical difference among the three groups in overall survival (OS), disease-free interval (DFI), and progress-free interval (PFI) comparisons (**Figure 2D**).

### HMOX1 is associated with novel genomic alterations in LUAD

**Figure 3:**
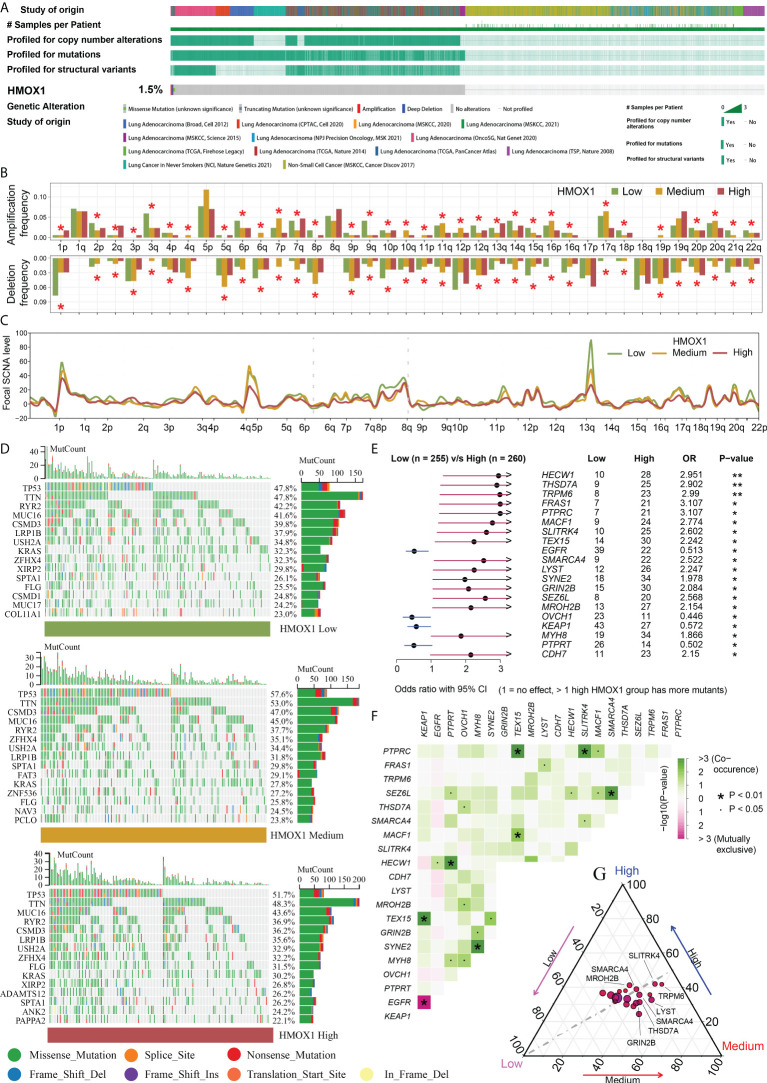
Genomic features of different HMOX1 groups. **(A)** The frequency of HMOX1 copy number alterations (CNAs) and mutations in the cBioPortal database. **(B)** Comparison of arm-level amplification and deletion frequencies among the high, medium, and low HMOX1 groups. **(C)** Comparison of focal-level amplification and deletion frequencies among the high, medium, and low HMOX1 groups. **(D)** The waterfall plots showing the mutation landscapes in the high, medium, and low HMOX1 groups. **(E)** The forest plot listing the top 20 most differentially mutated genes between the high and low HMOX1 groups. **(F)** The heatmap showing the concurrence or mutual exclusivity of these 20 mutated genes between the high and low HMOX1 groups. **(G)** The ternary plot showing the mutation frequency distribution of these 20 genes among the high, medium, and low HMOX1 groups. *P < 0.05; **P < 0.01.

cBioPortal data showed that the frequency of HMOX1 CNAs and mutations was only 1.5% in the LUAD (**Figure 3A**). Therefore, we did not focus on self-genomic alterations of HMOX1, and investigated the HMOX1-related alterations in the high, medium, and low HMOX1 groups. A global arm-and focal-level CNA profile was obtained by comparing the three groups (**Figures 3B, C**). The enriched mutation landscape of the three groups was displayed in waterfall plots. The missense mutation was the predominant mutation type (**Figure 3D**). Next, we compared the differentially mutated genes between the high and low HMOX1 groups. High HMOX1 groups had significantly higher frequency of mutations to HECW1, THSD7A, TRPM6, FRAS1, PTPRC, and others. Low HMOX1 groups had significantly higher frequency of mutations in EGFR, OVCH1, KEAP1, and PTPRT (**Figure 3E**). The strongest co-occurrent pairs of gene mutation in the high HMOX1 group were PTPRC - TEX15 and PTPRC - SLITRK4 (**Figure 3F**). In addition, we displayed the mutation frequency distribution of these genes in the three groups (**Figure 3G**).

### HMOX1-high LUAD is functionally enriched in immune and mitochondrial pathways

To explore other HMOX1-related phenotypes that may affect LUAD metastasis, we performed functional enrichment for DEGs between the high- and low-HMOX1 groups. In the GO analysis, DEGs were notably enriched in macrophage activation, macrophage migration, regulation of mitochondrion organization, apoptotic mitochondrial changes, and similar pathways (**Supplemental Figure 2A**). Among KEGG pathways, the most enriched were phagosome, Th17 cell differentiation, antigen processing and presentation, and others (**Supplemental Figure 2B**). Therefore, we investigated immune infiltration and mitochondrial processes in LUAD.

### HMOX1 is correlated with immune infiltration and immune checkpoint in LUAD

**Figure 4:**
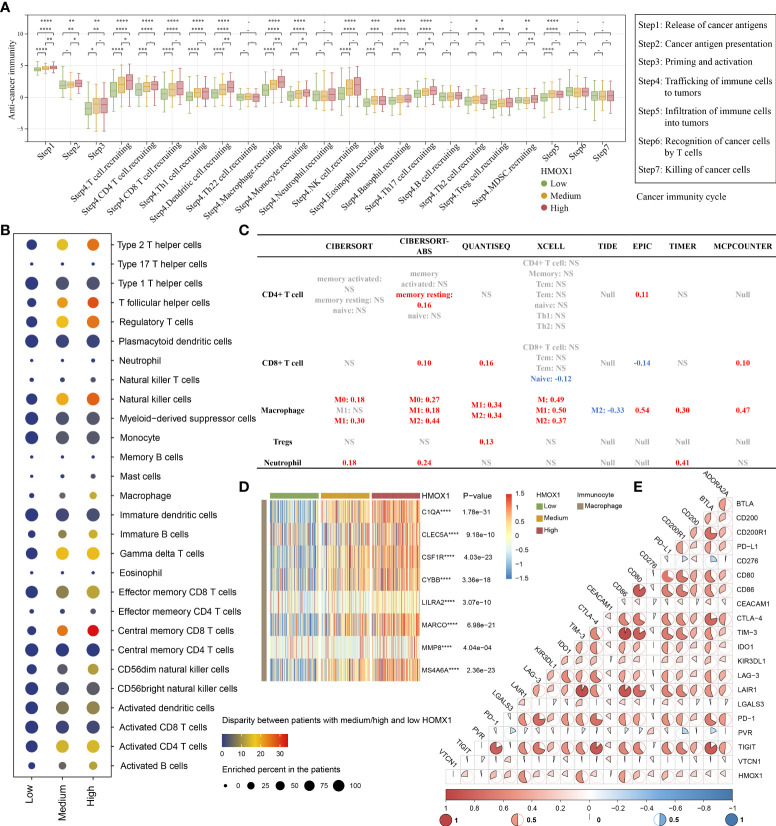
The effect of HMOX1 on the immune microenvironment in LUAD. **(A)** Differences in multiple steps of the cancer immunity cycles between the high, medium, and low HMOX1 groups. **(B)** Differences in the infiltration levels of 28 types of immunocytes among the high, medium, and low HMOX1 groups, which were calculated using the GSEA algorithms. **(C)** Correlation between HMOX1 and the infiltration levels of 5 types of immunocytes (CD4+ T cells, CD8+ T cells, macrophages, Tregs, and neutrophils), which were calculated using 8 algorithms. **(D)** Differences in the effector genes of macrophages among the high, medium, and low HMOX1 groups. **(E)** Correlation between HMOX1 and 20 inhibitory immune checkpoints. NS, not significant; *P < 0.05; **P < 0.01; ***P < 0.001; ****P < 0.0001.

For the high HMOX1 group, activities of the majority of the cancer-immune cycles were found to be up-regulated, including release of cancer antigens (step1), cancer antigen presentation (step 2), priming and activation (step 3), trafficking of immune cells to tumors (step 4, like CD4 T cell, CD8 T cell, macrophage, monocyte, Treg cell), and infiltration of immune cells into tumors (step 5). Interestingly, no statistical difference in the killing of cancer cells (step 7) was observed among the three HMOX1 groups (**Figure 4A**). Next, we calculated the level of immunocyte infiltration in LUAD by 9 algorithms. GSEA algorithms of TCIA showed that more immune cells, like type 2 T helper cells, regulatory T cells, macrophages, central memory CD8 T cells, activated CD4 T cells, and others were up-regulated in the high HMOX1 group, followed by the medium- and low-groups (**Figure 4B**). Another 8 algorithms showed that HMOX1 was positively associated with the infiltration level of macrophages, but not CD4 T cells, CD8 T cells, Tregs, or neutrophils (**Figure 4C**, and **Supplemental Figures 3**-**7**). Similarly, HMOX1 was found to be positively correlated to the effector genes of macrophages (**Figure 4D**). In addition, we found that HMOX1 expression was positively associated with a majority of immune checkpoint inhibitors, including LAIR1, TIM-3, CD86, and PD-L1 (**Figure 4E**).

### Mitochondrional complexes are altered in HMOX1 high LUAD

**Figure 5:**
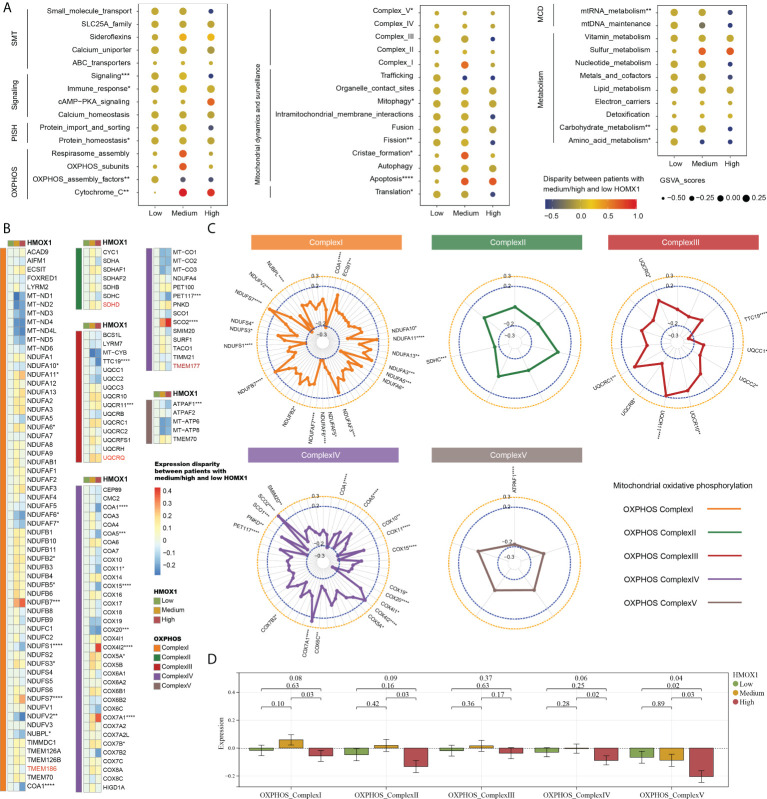
The effect of HMOX1 on mitochondrional activities in LUAD. **(A)** Differences in multiple mitochondrional pathways among the high, medium, and low HMOX1 groups. **(B)** Differences in feature genes of mitochondrial complexes among the high, medium, and low HMOX1 groups. **(C)** Correlation between HMOX1 and feature genes of mitochondrional complexes. **(D)** Differences in mitochondrional complex scoring between the high, medium, and low HMOX1 groups. SMT, small molecule transport; PISH, protein import, sorting and homeostasis; OXPHOS, oxidative phosphorylation; MCD, mitochondrial central dogma. *P < 0.05; **P < 0.01; ***P < 0.001; ****P < 0.0001.

We then proceeded to investigate the correlation between mitochondrial characteristics and HMOX1 expression in LUAD. Followed by the medium and low HMOX1 groups, the high HMOX1 group had significantly up-regulated expression of cytochrome C and apoptosis, and down-regulated expression of OXPHOS assembly factors, complexV, fission, translation, mtRNA metabolism, carbohydrate metabolism, and others (**Figure 5A**). Owing to the crucial role of mitochondrial complexes in tumor metastasis (8), we next focused on the analysis of mitochondrial complexes. The heatmap showed the expression disparity of feature genes for five mitochondrial complexes in the three HMOX1 groups (**Figure 5B**). The radar diagrams display the correlation between the feature gene and HMOX1 expression (**Figure 5C**). Overall, the high HMOX1 group had significantly less expression of complex I, complex II, and complex IV compared to the medium group, and significantly less expression of complex V compared to the low and medium group (**Figure 5D**).

### Construction of an HMOX1-mediated macrophage-mitochondrion-EMT metastasis regulatory network

**Figure 6:**
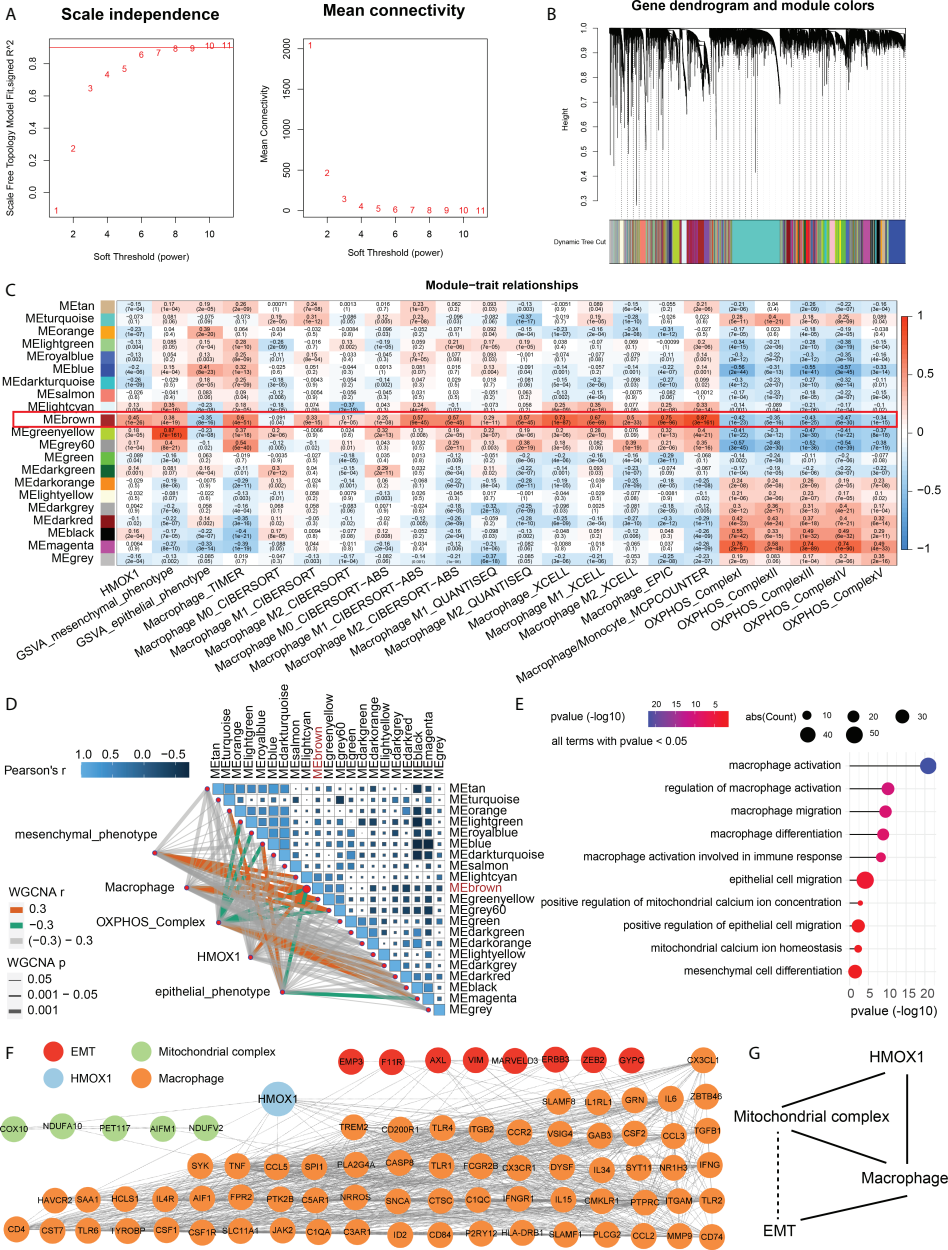
Weighted gene coexpression network analysis (WGCNA) for the construction of an HMOX1-mediated metastasis regulatory network in LUAD. **(A)** A scale-free network construction (power threshold β = 6). **(B)** Gene dendrogram generating gene modules. **(C, D)** Correlation analysis between modules and pathophysiological traits. HMOX1, EMT, macrophage, and mitochondrional complex had the same high correlation module (MEbrwon). **(E)** Gene Ontology (GO) enrichment for the MEbrown genes. **(F)** The construction of protein-protein interaction (PPI) network among HMOX1, macrophage, mitochondrional complex, and EMT using the MEbrown module genes. **(G)** The simplified interaction network among HMOX1, macrophage, mitochondrional complex, and EMT. Solid lines indicate interactions. Dashed lines indicate missing interactions.

Considering that mitochondrion complex, macrophage, and metastasis are all related to HMOX1 expression, we sought to perform the WGCNA co-expression analysis to identify the relationship among these. A power β = 6 was selected as the software threshold for scale-free network construction (**Figure 6A**). Twenty-one modules were identified by clustering dendrogram (**Figure 6B**). HMOX1, EMT, macrophage, and the mitochondrial complex had the same highly correlated module – brown, indicating strong associations among these traits (**Figure 6C**). The brown module was positively associated with HMOX1, mesenchymal phenotype, and macrophages, and negatively correlated to epithelial phenotype and mitochondrial complex (**Figure 6D**). GO enrichment indicated that genes in the brown module mainly focused on macrophage activation, macrophage migration, mitochondrial calcium ion homeostasis, mesenchymal cell differentiation, and the like (**Figure 6E**).

After identifying the correlation, we used the brown module genes to construct a PPI network among these phenotype traits. In the HMOX1-mediated metastasis regulatory network, a total of 8 genes were involved in EMT, 5 were correlated to mitochondrial complex, and 67 were associated with macrophage (**Figure 6F**). By analyzing the PPI interaction, we found that HMOX1 connected the macrophage and mitochondrial complex, the mitochondrial complex interacted with macrophage, and macrophage linked to EMT. However, the interaction between EMT and mitochondrial complex was not observed. (**Figure 6G**).

### Proteomic analysis for the HMOX1-mediated macrophage-mitochondrion-EMT metastasis regulatory network

**Figure 7:**
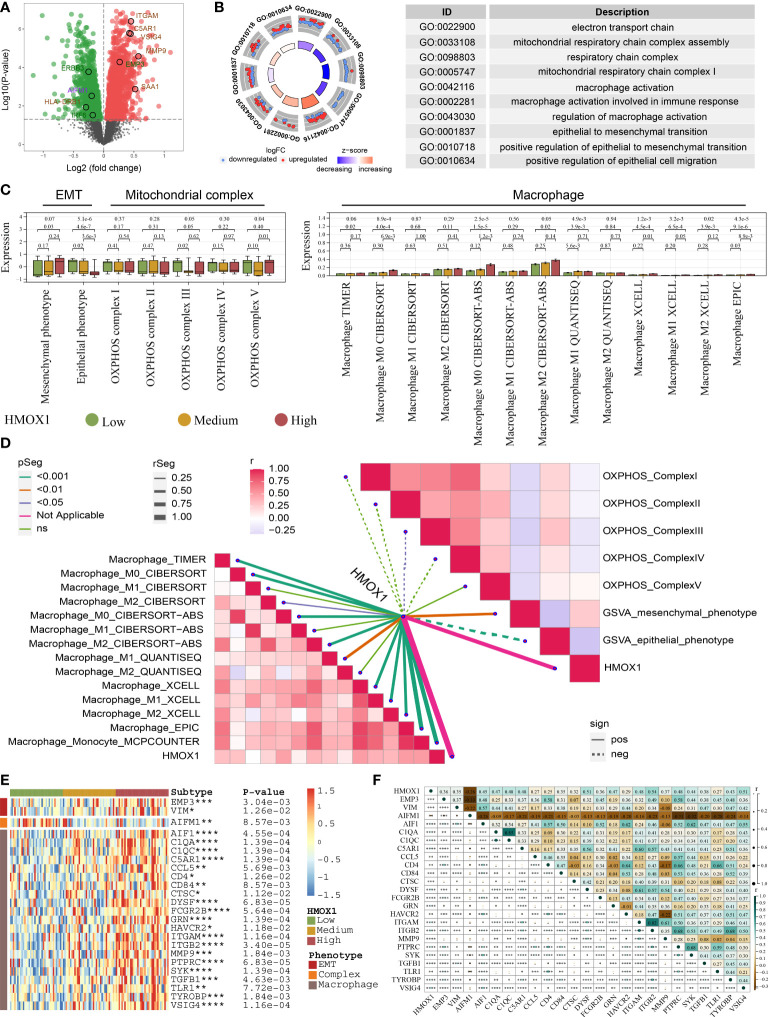
Proteomic validation for the HMOX1-mediated metastasis regulatory network in LUAD. **(A)** Volcano plots showing the differentially expressed proteins between the high and low HMOX1 groups. **(B)** GO enrichment for the differentially expressed proteins. **(C)** The difference in EMT, mitochondrial complex, and macrophage between the high, medium, and low HMOX1 groups. **(D)** Correlation between HMOX1 and EMT, mitochondrial complex, macrophage. **(E)** The difference in proteins of the HMOX1 regulatory network among high, medium, and low HMOX1 groups. **(F)** Correlation among proteins of the HMOX1 regulatory network *P < 0.05; **P < 0.01; ***P < 0.001; ****P < 0.0001.

By comparing the high and low HMOX1 group, 1481 down-regulated proteins and 1559 up-regulated proteins were obtained (**Figure 7A**). GO analysis showed that these differentially expressed proteins were enriched in the electron transport chain, mitochondrial respiratory chain complex assembly, macrophage activation, epithelial to mesenchymal transition, and similar pathways (**Figure 7B**). Next, we validated the relationship between HMOX1 and EMT, mitochondrial complex, and macrophage at the protein level. HMOX1 was positively associated with macrophage and mesenchymal phenotype, and negatively correlated to epithelial phenotype and mitochondrial complex III (**Figures 7C, D**). Furthermore, we analyzed the genes of the regulatory network at the protein level. The high HMOX1 group had higher protein expression of mesenchymal phenotype and macrophage, and lower protein expression of mitochondrial complexes, followed by the medium and low HMOX1 group (**Figure 7E**). Correlation analysis found that mitochondrial complex protein AIFM1 was dramatically negatively correlated to the protein expression of mesenchymal phenotype and macrophage (**Figure 7F**).

### Prognostic significance for the HMOX1-mediated macrophage-mitochondrion-EMT metastasis regulatory network

**Figure 8:**
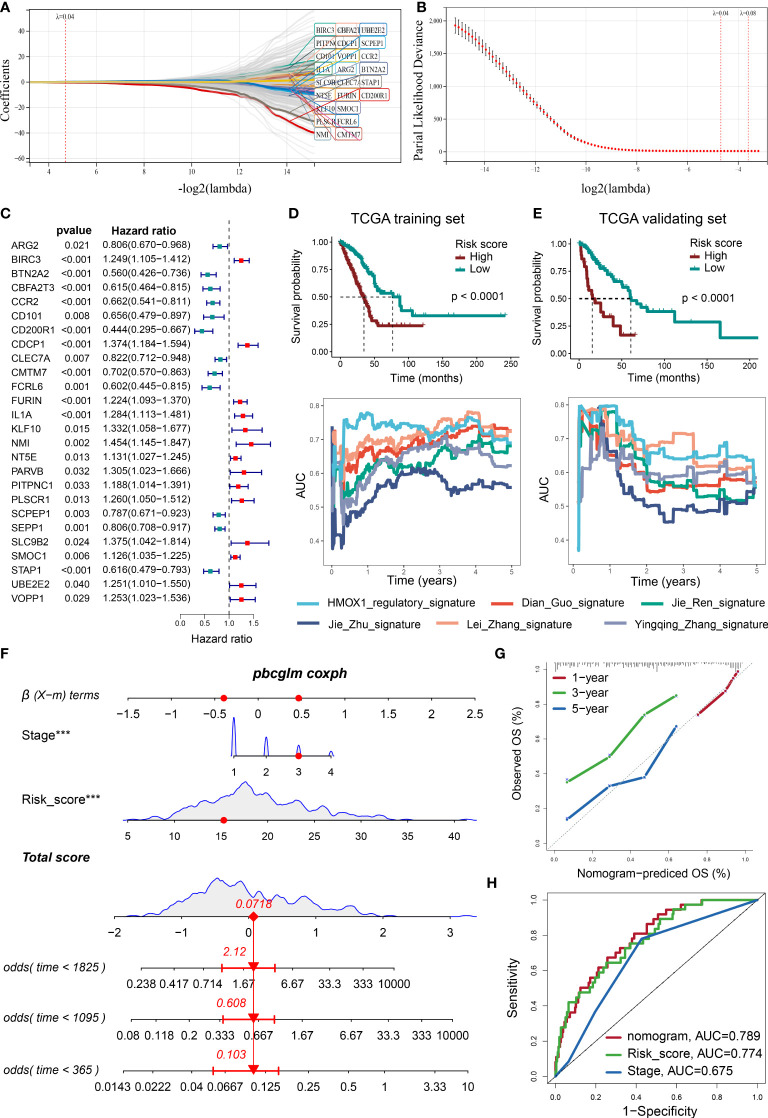
Developing a prognosis prediction model using the LASSO COX regression. **(A)** LASSO coefficient profiles of 307 prognosis-related genes, which were selected from the HMOX1-mediated metastasis regulatory network and its neighbor genes. **(B)** Cross-validation for tuning parameter screening in the LASSO regression model. Optimal genes with the best discriminative capability (26 in number) were selected for developing the risk scores. **(C)** Forest plot of the risk score gene-expression profiles in univariate analysis. **(D)** Development of risk score in the TCGA training cohort and its predictive accuracy for survival in comparison to additional 5 signatures. **(E)** validation of risk score in the TCGA validating cohort. **(F)** A nomogram integrating pathological stage, risk score, and OS probability in the TCGA training cohort. **(G)** Calibration plots showing the correlation between actual and predicted OS rates in the TCGA training cohort. **(H)** ROC curve plot evaluating the nomogram model, risk score, and pathological stage in the TCGA training cohort ***P < 0.001.

Using LASSO and Cox algorithms, 26 regulatory-network-related genes were screened to have the best predictive ability for prognosis in LUAD (**Figures 8A–C**). A risk score was then developed according to the gene expression and Cox regression coefficients of these genes. As shown in **Figures 8D, E**, the high-risk group had a significantly worse prognosis than the low-risk group in the TCGA training and validating cohorts. External GEO validating cohorts also confirmed this result (**Supplemental Figure 8A**). The real-time AUC values of our risk score were also generally higher than similar prognostic scoring systems (Dian Guo (18), Jie Ren (19), Jie Zhu (20), Lei Zhang (21), Yingqing Zhang (22)) in LUAD (**Figures 8D, E**). Next, we identified the independent prognostic factors by Cox analysis, including risk score and pathological stage (**Supplemental Figures 8B, C**). A nomogram model was constructed using these factors (**Figure 8F**). Calibration plots indicated that observed and predicted probabilities for 1-, 3-, and 5-year overall survival (OS) had excellent concordance (**Figure 8G** and **Supplemental Figure 8D**). ROC curves further confirmed the excellent predictive power of the nomogram (AUC: 0.789 in training TCGA, 0.854 in validating TCGA 0.765 in GSE11969, 0.877 in GSE31210, 0.639 in GSE42127) in both training and validating cohorts (**Figure 8H** and **Supplemental Figure 8E**). Regrettably, GSE68486 could not be used to validate the nomogram model, owing to a lack of the variable for pathological grade.

### Prediction of novel targeting modalities for the HMOX1-mediated macrophage-mitochondrion-EMT metastasis regulatory network

To predict the drugs with the potential to inhibit HMOX1-mediated LUAD metastasis, we uploaded the regulatory network genes to the CMAP online tools. We identified 9 drugs (chloroxine, tetrabenazine, TPCA-1, aminopentamide, coumaric-acid, zosuquidar, lupanine, isoliquiritigenin, pilocarpine) with the negative Raw_cs and the top fdr_q_nlog10 values, which suggested they could inhibit the gene expression in the regulatory network (**Table 2** and **Supplemental Figure 9**).

**Table 2:** Identified 9 small molecular drugs by CMAP.

Rank	CMAP name	MOA	Raw_cs	fdr_q_nlog10
1	chloroxine	Opioid receptor antagonist	-0.6937	15.6536
2	tetrabenazine	Vesicular monoamine transporter inhibitor	-0.6785	15.6536
3	TPCA-1	IKK inhibitor	-0.6768	15.6536
4	aminopentamide	Acetylcholine receptor antagonist	-0.6756	15.6536
5	coumaric-acid	Antioxidant	-0.6734	15.6536
6	zosuquidar	P-glycoprotein inhibitor	-0.673	15.6536
7	lupanine	Sodium channel inhibitor	-0.6646	15.6536
8	isoliquiritigenin	Guanylate cyclase activator	-0.653	15.6536
9	Pilocarpine	Acetylcholine receptor agonist	-0.6476	15.6536

## Discussion

Emerging evidence has indicated the effect of HMOX1 in tumor metastasis, and HMOX1 is attractive as a potential prognostic biomarker and therapeutic target. Since the role of HMOX1 in LUAD metastasis still remains unclear, we performed large-scale and multi-omics bioinformatic analyses to elucidate HMOX1 in LUAD metastasis. In our study, we first demonstrated that HMOX1 contributed to the metastasis of multiple tumors, especially LUAD. Next, we found that HMOX1 was also correlated with the expression of macrophages and the mitochondrional complex. Importantly, by co-expression analysis, we identified a group of genes co-regulating the phenotypes mentioned above, and constructed the HMOX1-mediated macrophage-mitochondrion-EMT metastasis regulatory network. Based on the PPI network and existing studies, we speculated that HMOX1 may mediate the LUAD metastasis by regulating macrophages and the mitochondrional complex. Finally, the survival analysis and drug analysis were performed for HMOX1-mediated metastasis in LUAD.

HMOX1 has been found to be involved in the multiple steps of tumor metastasis including invasion, intravasation, extravasation, and colonization. Previous studies have indicated that the inhibition of HMOX1 reversed tumor EMT and impaired the invasion and migration of tumor cells (23). HMOX1 activity could also facilitate transendothelial migration of tumor cells, which implicates a potential role for HMOX1 in intravasation and extravasation events (24). In addition, myeloid HMOX1 promoted the extravasation and colonization of tumor cells at the metastatic loci (25). Similar to the previous studies, our results demonstrate that HMOX1 expression is positively associated with the EMT and lymph node and distant metastasis in multiple types of tumors, especially LUAD. The high HMOX1 group also had higher proportions of the squamoid subtype, which is a worse LUAD subtype with predilection for brain metastasis in the early stages (26). HMOX1-mediated LUAD metastasis has been preliminarily reported. Tsai *et al.* found that NSCLC patients with high HMOX1 expression had higher metastatic rates and HMOX1 over-overexpression enhanced the migratory ability of LUAD cells (27). However, the detailed mechanism of HMOX1-mediated LUAD metastasis still remains largely unknown.

Genomic alteration drives tumor metastasis (28). HMOX1 self-mutation is rare in LUAD. Therefore, we aimed to understand the HMOX1-related mutational burden and identified 16 differentially mutated genes in patients with high HMOX1 expression. Among these, FRAS1, PTPRC, MACF1 and MYH8 mutations have been found to be more enriched in other metastatic tumors (29–32). SMARCA4 mutation was reported to promote the LUAD early metastasis (33). SMARCA4-deficient LUAD had higher aggressive behavior and metastasis potential to the pleura (34). In addition, Kim *et al.* found that SMARCA4 depletion induced the EMT process in triple-negative breast cancer (35).

Macrophages, as a major immunocyte of the tumor microenvironment, play a vital role in tumor metastasis. The size, shape, metabolism, and M2 polarization of macrophages affect their metastasis (36, 37). At the primary site, macrophages can activate the EMT process and increase tumor cell migration and invasion (7, 38). At the metastatic loci, macrophages can prepare the target tissue for the arrival of tumor cells, and promote tumor cell extravasation, survival, and subsequent growth (38). Our result showed that HMOX1 was positively associated with macrophage recruitment, expression, and activity in LUAD. Similarly, some studies have demonstrated that HMOX1 was critically involved in macrophage polarization (12). In addition, a recent study has identified a novel macrophage subtype, endowed with high heme catabolism by HMOX1, as a pro-metastatic tumor microenvironment remodeling factor favoring EMT (39). Therefore, we retained the macrophage as the candidate constituent for the HMOX1 metastasis regulatory network.

The mitochondria supply the energy for tumor cell migration, invasion, and metastasis (40). Five complexes of the mitochondrial electron transport chain can affect tumor metastasis (8). Emergent evidence indicates that down-modulation of certain complex subunits by genetic or pharmacologic may promote EMT and metastasis (8, 41–43). In our study, we found down-regulated expression of mitochondrional complexes in the high HMOX1 group at the RNA and protein levels. Previous studies have reported the close correlation between HMOX1 and mitochondrion. Bansal *et al.* showed that mitochondria-targeted HMOX1 could induce mitochondrial dysfunction in macrophages (44). Song *et al.* found that HMOX1 up-regulation enhanced the oxidative mitochondrial damage in astroglia (45). Therefore, we speculate that the mitochondrion complex may affect the HMOX1-mediated metastasis in LUAD.

To further identify the correlation between the HMOX1, metastasis, macrophages, and mitochondrion, we performed the WGCNA analysis and found a group of module genes co-regulating these traits. The module genes had a highly positive correlation with HMOX1, mesenchymal phenotype and macrophages, and a highly negative association with epithelial phenotype, and mitochondrial complexes. Based on the module genes, we constructed an HMOX1-mediated macrophage-mitochondrion-EMT metastasis regulatory network. The network showed a high inner correlation at the proteome level and high predictive ability for survival prognosis in LUAD patients. According to the network and existing studies, we speculate that HMOX1 may mediate the EMT and further LUAD metastasis by regulating mitochondrial complex and macrophage. Interestingly, the network lacks the connection between mitochondrial complex and EMT. Considering the EMT process needs large amounts of energy from the mitochondrion, we speculate that the missing connection may be attributed to the low number of studies on the interaction between mitochondrial complexes and EMT, which deserves further study.

Previous studies have also identified the association between mitochondrion and macrophages. Many mitochondria activities, including tricarboxylic acid cycle, oxidative metabolism, membrane potential, and others, are vital for macrophage activation, differentiation, and survival (46). Among these, mitochondrial fission-induced mtDNA stress could promote tumor-associated macrophage infiltration and ultimately lead to tumor progression (47). Targeting mitochondrial complex I and blocking macrophage-mediated adaptive responses could play a synergistic role to induce cancer indolence (48).

Pharmacological inhibition of HMOX1 is considered a promising therapeutic approach to hinder tumor metastasis. In this research, we predicted nine small molecule drugs targeting the HMOX1-mediated metastasis regulatory network. Among these, chloroxine, belonging to opioid receptor antagonists, has been reported to inhibit tumor metastasis. Chloroxine-inducible Par-4 secretion could induce tumor cell apoptosis and impede tumor metastasis (49). Polymeric chloroquine was also confirmed to suppress the lung metastasis of tumor cells (50). In addition, isoliquiritigenin, a guanylate cyclase activator, has been proven to suppress metastasis in multiple types of tumors, including melanoma (51), breast cancer (52), liver cancer (53), renal cell carcinoma (54), and others. Of note, a previous study showed that isoliquiritigenin reversed the EMT process to inhibit ovarian cancer metastasis (55).

In conclusion, our analyses elaborate on the role of HMOX1 in LUAD metastasis, and identify an HMOX1-mediated macrophage-mitochondrion-EMT metastasis regulatory network. Furthermore, a network-related prognostic model was established, and potential drugs targeting the HMOX1 regulatory network were also proposed, which may inhibit LUAD metastasis. One major limitation was the lack of a single-cell location analysis for HMOX1 expression. Another limitation was the lack of intercellular interaction analysis between macrophage and malignant tumor cells, which we expect to improve in subsequent studies. Additionally, the regulatory network will require further experimental validation.

## Data availability statement

The original contributions presented in the study are included in the article/**Supplementary Material**. Further inquiries can be directed to the corresponding authors.

## Ethics statement

This study has been approved by the Medical Ethics committee of the Xiangya Hospital of Central South University (No. 202012388 & No. 2017121019) and the patients/participants have provided their written informed consent.

## Author contributions

AT designed the research. AT, BC, and LZ drafted the manuscript. BC, LZ, HZ, AT, WY and CL organized figures and figure legends. WY, CL, NF and AT revised the article. BC, LZ and HZ conducted data analysis. All authors contributed to the article and approved the submitted version.

## Funding

This work was supported by the Nature Science Foundation of China (No. 81402249 to LZ), the Natural Science Foundation of Hunan Province (No. 2019JJ50963 to LZ), and Fundamental Research Funds for the Central Universities of Central South University (No. 160171016 to BC).

## Conflict of interest

The authors declare that the research was conducted in the absence of any commercial or financial relationships that could be construed as a potential conflict of interest.

## Publisher’s note

All claims expressed in this article are solely those of the authors and do not necessarily represent those of their affiliated organizations, or those of the publisher, the editors and the reviewers. Any product that may be evaluated in this article, or claim that may be made by its manufacturer, is not guaranteed or endorsed by the publisher.

## Supplementary material

Supplementary Figure 1 The flow chart of data analysis.

Supplementary Figure 2 Function enrichment between the high and low HMOX1 groups. **(A)** Go enrichment for the differentially expressed genes (DEGs) between the high and low HMOX1 groups. **(B)** Kyoto Encyclopedia of Genes and Genomes (KEGG) enrichment for the DEGs between the high and low HMOX1 groups.

Supplementary Figure 3 Correlation between HMOX1 and the infiltration levels of immunocytes, including B cell **(A)** and dendritic cell (DC, **B**).

Supplementary Figure 4 Correlation between HMOX1 and the infiltration levels of immunocytes, including macrophage **(A)** and T cell CD8+ **(B)**.

Supplementary Figure 5 Correlation between HMOX1 and the infiltration levels of immunocytes, including T cell CD4+ **(A)**, Eosinphil **(B)**. and mast cell **(C)**.

Supplementary Figure 6 Correlation between HMOX1 and the infiltration levels of immunocytes, including monocyte **(A)**, endothelial cell **(B)**, neutrophil cell **(C)**, T cell gamma delta **(D)**, T cell NK **(E)**, hematopoietic stem cell **(F)**, and myeloid-derived suppressor cell **(**MDCS, **G)**.

Supplementary Figure 7 Correlation between HMOX1 and the infiltration levels of immunocytes, including NK cell **(A)**, common myeloid progenitor **(**GMP, **B)**, cancer-associated fibroblast **(C)**, T cell regulatory **(D)**, T cell follicular helper (E), common lymphoid progenitor **(**CLP, **F)**, and common myeloid progenitor **(**CMP, **G)**.

Supplementary Figure 8 Validation of the nomogram prognosis model in the TCGA validating and GEO cohorts. **(A)** Comparison of survival curves between the high and low HMOX1 groups in the GSE11969, GSE31210, GSE42127, and GSE68465 cohorts. **(B)** Univariate Cox analysis screened the prognosis variables. **(C)** Multiple Cox analysis screened the prognosis variables. **(D)** Calibration plots showing the correlation between actual and predicted OS rates in the TCGA validating, GSE11969, GSE31210, and GSE42127 cohorts. **(E)** ROC curve plot evaluating the nomogram model in the TCGA, GSE11969, GSE31210, and GSE42127 validating cohort.

Supplementary Figure 9 Identifying the molecular structure of 9 small component drugs targeting the HMOX1-mediated LUAD metastasis by CMAP.

## Abbreviations

Clinical Proteomic Tumor Analysis Consortium, CPTAC; Connectivity Map, CMAP; Copy number alternation, CNA; Differentially expressed gene, DEG; Disease-free interval, DFI; Disease-specific survival = DSS; Epithelial-mesenchymal transition, EMT; Gene Expression Omnibus, GEO; Gene Ontology, GO; Gene Set Variation Analysis, GSVA; Heme oxygenase-1, HMOX1; Human Cancer Metastasis Database, HCMDB; Kyoto Encyclopedia of Genes and Genomes, KEGG; Least absolute shrinkage and selection operator, Lasso; Lung adenocarcinoma, LUAD; Non-small cell lung cancer, NSCLC; Non-small cell lung cancer, NSCLC; Overall survival, OS; Progress-free interval, PFI; Prostate adenocarcinoma, PRAD; Receiver operating characteristic, ROC; Small cell lung cancer, SCLC; The Cancer Genome Atlas, TCGA; The Cancer Immunome Atlas, TCIA; Weighted gene co-expression network analysis, WGCNA

## Glossary

**Table:**

CPTAC	Clinical Proteomic Tumor Analysis Consortium
CMAP	Connectivity Map
CNA	Copy number alternation
DEG	Differentially expressed gene
DFI	Disease-free interval
DSS	Disease-specific survival
EMT	Epithelial-mesenchymal transition
GEO	Gene Expression Omnibus
GO	Gene Ontology
GSVA	Gene Set Variation Analysis
HMOX1	Heme oxygenase-1
HCMDB	Human Cancer Metastasis Database
KEGG	Kyoto Encyclopedia of Genes and Genomes
Lasso	Least absolute shrinkage and selection operator
LUAD	Lung adenocarcinoma
NSCLC	Non-small cell lung cancer
NSCLC	Non-small cell lung cancer
OS	Overall survival
PFI	Progress-free interval
PRAD	Prostate adenocarcinoma
ROC	Receiver operating characteristic
SCLC	Small cell lung cancer
TCGA	The Cancer Genome Atlas
TCIA	The Cancer Immunome Atlas
WGCNA	Weighted gene co-expression network analysis

## References

[1] Duma N Santana-Davila R Molina JR. Non-small cell lung cancer: Epidemiology, screening, diagnosis, and treatment. Mayo Clin Proc (2019) 94(8):1623–40. doi: 10.1016/j.mayocp.2019.01.013 31378236

[2] Ettinger DS Wood DE Aisner DL Akerley W Bauman JR Bharat A. NCCN guidelines insights: Non-small cell lung cancer, version 2.2021. J Natl Compr Canc Netw (2021) 19(3):254–66. doi: 10.6004/jnccn.2021.0013 33668021

[3] Riihimäki M Hemminki A Fallah M Thomsen H Sundquist K Sundquist J. Metastatic sites and survival in lung cancer. Lung Cancer (2014) 86(1):78–84. doi: 10.1016/j.lungcan.2014.07.020 25130083

[4] Fares J Fares MY Khachfe HH Salhab HA Fares Y. Molecular principles of metastasis: a hallmark of cancer revisited. Signal Transduct Target Ther (2020) 5(1):28. doi: 10.1038/s41392-020-0134-x 32296047 PMC7067809

[5] Kalluri R Weinberg RA. The basics of epithelial-mesenchymal transition. J Clin Invest (2009) 119(6):1420–8. doi: 10.1172/jci39104 PMC2689101 19487818

[6] Wang G Xu D Zhang Z Li X Shi J Sun J. The pan-cancer landscape of crosstalk between epithelial-mesenchymal transition and immune evasion relevant to prognosis and immunotherapy response. NPJ Precis Oncol (2021) 5(1):56. doi: 10.1038/s41698-021-00200-4 34158591 PMC8219790

[7] El-Kenawi A Hänggi K Ruffell B. The immune microenvironment and cancer metastasis. Cold Spring Harb Perspect Med (2020) 10(4):a037424. doi: 10.1101/cshperspect.a037424 31501262 PMC7117953

[8] Urra FA Muñoz F Lovy A Cárdenas C. The mitochondrial Complex(I)ty of cancer. Front Oncol (2017) 7:118. doi: 10.3389/fonc.2017.00118 28642839 PMC5462917

[9] Luu Hoang KN Anstee JE Arnold JN. The diverse roles of heme oxygenase-1 in tumor progression. Front Immunol (2021) 12:658315. doi: 10.3389/fimmu.2021.658315 33868304 PMC8044534

[10] Shibahara S Müller R Taguchi H Yoshida T. Cloning and expression of cDNA for rat heme oxygenase. Proc Natl Acad Sci U.S.A. (1985) 82(23):7865–9. doi: 10.1073/pnas.82.23.7865 PMC390870 3865203

[11] Bindu S Pal C Dey S Goyal M Alam A Iqbal MS. Translocation of heme oxygenase-1 to mitochondria is a novel cytoprotective mechanism against non-steroidal anti-inflammatory drug-induced mitochondrial oxidative stress, apoptosis, and gastric mucosal injury. J Biol Chem (2011) 286(45):39387–402. doi: 10.1074/jbc.M111.279893 PMC3234763 21908612

[12] Weis N Weigert A von Knethen A Brüne B. Heme oxygenase-1 contributes to an alternative macrophage activation profile induced by apoptotic cell supernatants. Mol Biol Cell (2009) 20(5):1280–8. doi: 10.1091/mbc.e08-10-1005 PMC2649271 19129475

[13] Higdon AN Benavides GA Chacko BK Ouyang X Johnson MS Landar A. Hemin causes mitochondrial dysfunction in endothelial cells through promoting lipid peroxidation: the protective role of autophagy. Am J Physiol Heart Circ Physiol (2012) 302(7):H1394–409. doi: 10.1152/ajpheart.00584.2011 PMC3330785 22245770

[14] Mak MP Tong P Diao L Cardnell RJ Gibbons DL William WN. A patient-derived, pan-cancer EMT signature identifies global molecular alterations and immune target enrichment following epithelial-to-Mesenchymal transition. Clin Cancer Res (2016) 22(3):609–20. doi: 10.1158/1078-0432.Ccr-15-0876 PMC4737991 26420858

[15] Hänzelmann S Castelo R Guinney J. GSVA: gene set variation analysis for microarray and RNA-seq data. BMC Bioinf (2013) 14:7. doi: 10.1186/1471-2105-14-7 PMC3618321 23323831

[16] Hu J Yu A Othmane B Qiu D Li H Li C. Siglec15 shapes a non-inflamed tumor microenvironment and predicts the molecular subtype in bladder cancer. Theranostics (2021) 11(7):3089–108. doi: 10.7150/thno.53649 PMC7847675 33537076

[17] Zampieri LX Silva-Almeida C Rondeau JD Sonveaux P. Mitochondrial transfer in cancer: A comprehensive review. Int J Mol Sci (2021) 22(6):3245. doi: 10.3390/ijms22063245 33806730 PMC8004668

[18] Guo D Wang M Shen Z Zhu J. A new immune signature for survival prediction and immune checkpoint molecules in lung adenocarcinoma. J Transl Med (2020) 18(1):123. doi: 10.1186/s12967-020-02286-z 32143735 PMC7060601

[19] Ren J Zhang H Wang J Xu Y Zhao L Yuan Q. Transcriptome analysis of adipocytokines and their-related LncRNAs in lung adenocarcinoma revealing the association with prognosis, immune infiltration, and metabolic characteristics. Adipocyte (2022) 11(1):250–65. doi: 10.1080/21623945.2022.2064956 PMC9037474 35410586

[20] Zhu J Wang M Hu D. Development of an autophagy-related gene prognostic signature in lung adenocarcinoma and lung squamous cell carcinoma. Peer J (2020) 8:e8288. doi: 10.7717/peerj.8288 31938577 PMC6953332

[21] Zhang L Zhang Z Yu Z. Identification of a novel glycolysis-related gene signature for predicting metastasis and survival in patients with lung adenocarcinoma. J Transl Med (2019) 17(1):423. doi: 10.1186/s12967-019-02173-2 31847905 PMC6916245

[22] Zhang Y Zhang X Lv X Zhang M Gao X Liu J. Development and validation of a seven-gene signature for predicting the prognosis of lung adenocarcinoma. BioMed Res Int (2020) 2020:1836542. doi: 10.1155/2020/1836542 33195688 PMC7641279

[23] Zhao Z Zhao J Xue J Zhao X Liu P. Autophagy inhibition promotes epithelial-mesenchymal transition through ROS/HO-1 pathway in ovarian cancer cells. Am J Cancer Res (2016) 6(10):2162–77. PMC5088283 27822409

[24] Muliaditan T Caron J Okesola M Opzoomer JW Kosti P Georgouli M. Macrophages are exploited from an innate wound healing response to facilitate cancer metastasis. Nat Commun (2018) 9(1):2951. doi: 10.1038/s41467-018-05346-7 30054470 PMC6063977

[25] Lin HH Chiang MT Chang PC Chau LY. Myeloid heme oxygenase-1 promotes metastatic tumor colonization in mice. Cancer Sci (2015) 106(3):299–306. doi: 10.1111/cas.12604 25580731 PMC4376439

[26] Hayes DN Monti S Parmigiani G Gilks CB Naoki K Bhattacharjee A. Gene expression profiling reveals reproducible human lung adenocarcinoma subtypes in multiple independent patient cohorts. J Clin Oncol (2006) 24(31):5079–90. doi: 10.1200/jco.2005.05.1748 17075127

[27] Tsai JR Wang HM Liu PL Chen YH Yang MC Chou SH. High expression of heme oxygenase-1 is associated with tumor invasiveness and poor clinical outcome in non-small cell lung cancer patients. Cell Oncol (Dordr) (2012) 35(6):461–71. doi: 10.1007/s13402-012-0105-5 PMC12995167 23055342

[28] Nguyen B Fong C Luthra A Smith SA DiNatale RG Nandakumar S. Genomic characterization of metastatic patterns from prospective clinical sequencing of 25,000 patients. Cell (2022) 185(3):563–75.e11. doi: 10.1016/j.cell.2022.01.003 35120664 PMC9147702

[29] Lefebvre C Bachelot T Filleron T Pedrero M Campone M Soria JC. Mutational profile of metastatic breast cancers: A retrospective analysis. PloS Med (2016) 13(12):e1002201. doi: 10.1371/journal.pmed.1002201 28027327 PMC5189935

[30] Alanazi IO Alamery SF Ebrahimie E Mohammadi-Dehcheshmeh M. Splice-disrupt genomic variants in prostate cancer. Mol Biol Rep (2022) 49(6):4237–46. doi: 10.1007/s11033-022-07257-9 PMC9262760 35286517

[31] Chen C Shi C Huang X Zheng J Zhu Z Li Q. Molecular profiles and metastasis markers in Chinese patients with gastric carcinoma. Sci Rep (2019) 9(1):13995. doi: 10.1038/s41598-019-50171-7 31570735 PMC6769015

[32] Park H Kim D Kim D Park J Koh Y Yoon SS. Truncation of MYH8 tail in AML: a novel prognostic marker with increase cell migration and epithelial-mesenchymal transition utilizing RAF/MAPK pathway. Carcinogenesis (2020) 41(6):817–27. doi: 10.1093/carcin/bgz146 31430364

[33] Concepcion CP Ma S LaFave LM Bhutkar A Liu M DeAngelo LP. Smarca4 inactivation promotes lineage-specific transformation and early metastatic features in the lung. Cancer Discovery (2022) 12(2):562–85. doi: 10.1158/2159-8290.Cd-21-0248 PMC8831463 34561242

[34] Armon S Hofman P Ilié M. Perspectives and issues in the assessment of SMARCA4 deficiency in the management of lung cancer patients. Cells (2021) 10(8):1920. doi: 10.3390/cells10081920 34440689 PMC8394288

[35] Kim J Jang G Sim SH Park IH Kim K Park C. SMARCA4 depletion induces cisplatin resistance by activating YAP1-mediated epithelial-to-Mesenchymal transition in triple-negative breast cancer. Cancers (Basel) (2021) 13(21):5474. doi: 10.3390/cancers13215474 34771636 PMC8582548

[36] Murray PJ. Cancer metastasis linked to macrophage size, shape, and metabolism. J Exp Med (2020) 217(11):e20201259. doi: 10.1084/jem.20201259 32997733 PMC7596826

[37] Zhao S Mi Y Guan B Zheng B Wei P Gu Y. Tumor-derived exosomal miR-934 induces macrophage M2 polarization to promote liver metastasis of colorectal cancer. J Hematol Oncol (2020) 13(1):156. doi: 10.1186/s13045-020-00991-2 33213490 PMC7678301

[38] Qian BZ Pollard JW. Macrophage diversity enhances tumor progression and metastasis. Cell (2010) 141(1):39–51. doi: 10.1016/j.cell.2010.03.014 20371344 PMC4994190

[39] Consonni FM Bleve A Totaro MG Storto M Kunderfranco P Termanini A. Heme catabolism by tumor-associated macrophages controls metastasis formation. Nat Immunol (2021) 22(5):595–606. doi: 10.1038/s41590-021-00921-5 33903766

[40] Gundamaraju R Lu W Manikam R. Revisiting mitochondria scored cancer progression and metastasis. Cancers (Basel) (2021) 13(3):432. doi: 10.3390/cancers13030432 33498743 PMC7865825

[41] Li J Liang N Long X Zhao J Yang J Du X. SDHC-related deficiency of SDH complex activity promotes growth and metastasis of hepatocellular carcinoma *via* ROS/NFκB signaling. Cancer Lett (2019) 461:44–55. doi: 10.1016/j.canlet.2019.07.001 31278950

[42] Li LD Sun HF Liu XX Gao SP Jiang HL Hu X. Down-regulation of NDUFB9 promotes breast cancer cell proliferation, metastasis by mediating mitochondrial metabolism. PloS One (2015) 10(12):e0144441. doi: 10.1371/journal.pone.0144441 26641458 PMC4671602

[43] Yuan Y Wang W Li H Yu Y Tao J Huang S. Nonsense and missense mutation of mitochondrial ND6 gene promotes cell migration and invasion in human lung adenocarcinoma. BMC Cancer (2015) 15:346. doi: 10.1186/s12885-015-1349-z 25934296 PMC4425906

[44] Bansal S Biswas G Avadhani NG. Mitochondria-targeted heme oxygenase-1 induces oxidative stress and mitochondrial dysfunction in macrophages, kidney fibroblasts and in chronic alcohol hepatotoxicity. Redox Biol (2014) 2:273–83. doi: 10.1016/j.redox.2013.07.004 PMC3909819 24494190

[45] Song W Su H Song S Paudel HK Schipper HM. Over-expression of heme oxygenase-1 promotes oxidative mitochondrial damage in rat astroglia. J Cell Physiol (2006) 206(3):655–63. doi: 10.1002/jcp.20509 16222706

[46] Wang Y Li N Zhang X Horng T. Mitochondrial metabolism regulates macrophage biology. J Biol Chem (2021) 297(1):100904. doi: 10.1016/j.jbc.2021.100904 34157289 PMC8294576

[47] Bao D Zhao J Zhou X Yang Q Chen Y Zhu J. Mitochondrial fission-induced mtDNA stress promotes tumor-associated macrophage infiltration and HCC progression. Oncogene (2019) 38(25):5007–20. doi: 10.1038/s41388-019-0772-z PMC6755992 30894684

[48] Kurelac I Iommarini L Vatrinet R Amato LB De Luise M Leone G. Inducing cancer indolence by targeting mitochondrial complex I is potentiated by blocking macrophage-mediated adaptive responses. Nat Commun (2019) 10(1):903. doi: 10.1038/s41467-019-08839-1 30796225 PMC6385215

[49] Burikhanov R Hebbar N Noothi SK Shukla N Sledziona J Araujo N. Chloroquine-inducible par-4 secretion is essential for tumor cell apoptosis and inhibition of metastasis. Cell Rep (2017) 18(2):508–19. doi: 10.1016/j.celrep.2016.12.051 PMC5264245 28076793

[50] Yu F Li J Xie Y Sleightholm RL Oupický D. Polymeric chloroquine as an inhibitor of cancer cell migration and experimental lung metastasis. J Controlled Release (2016) 244(Pt B):347–56. doi: 10.1016/j.jconrel.2016.07.040 PMC5167664 27473763

[51] Xiang S Zeng H Xia F Ji Q Xue J Ren R. The dietary flavonoid isoliquiritigenin induced apoptosis and suppressed metastasis in melanoma cells: An *in vitro* and *in vivo* study. Life Sci (2021) 264:118598. doi: 10.1016/j.lfs.2020.118598 33189818

[52] Peng F Tang H Du J Chen J Peng C. Isoliquiritigenin suppresses EMT-induced metastasis in triple-negative breast cancer through miR-200c/C-JUN/[Formula: see text]-catenin. Am J Chin Med (2021) 49(2):505–23. doi: 10.1142/s0192415x21500233 33641651

[53] Huang Y Liu C Zeng WC Xu GY Wu JM Li ZW. Isoliquiritigenin inhibits the proliferation, migration and metastasis of Hep3B cells *via* suppressing cyclin D1 and PI3K/AKT pathway. Biosci Rep (2020) 40(1):BSR20192727. doi: 10.1042/bsr20192727 31840737 PMC6944659

[54] Yamazaki S Morita T Endo H Hamamoto T Baba M Joichi Y. Isoliquiritigenin suppresses pulmonary metastasis of mouse renal cell carcinoma. Cancer Lett (2002) 183(1):23–30. doi: 10.1016/s0304-3835(02)00113-1 12049811

[55] Chen C Huang S Chen CL Su SB Fang DD. Isoliquiritigenin inhibits ovarian cancer metastasis by reversing epithelial-to-Mesenchymal transition. Molecules (2019) 24(20):3725. doi: 10.3390/molecules24203725 PMC6833095 31623144

