# Supplementary figures and images for: HMOX1 promotes lung adenocarcinoma metastasis by affecting macrophages and mitochondrion complexes
Source: Front Oncol. 2022 Aug 12;12:978006. doi: 10.3389/fonc.2022.978006 (PMC9417688; doi:10.3389/fonc.2022.978006)

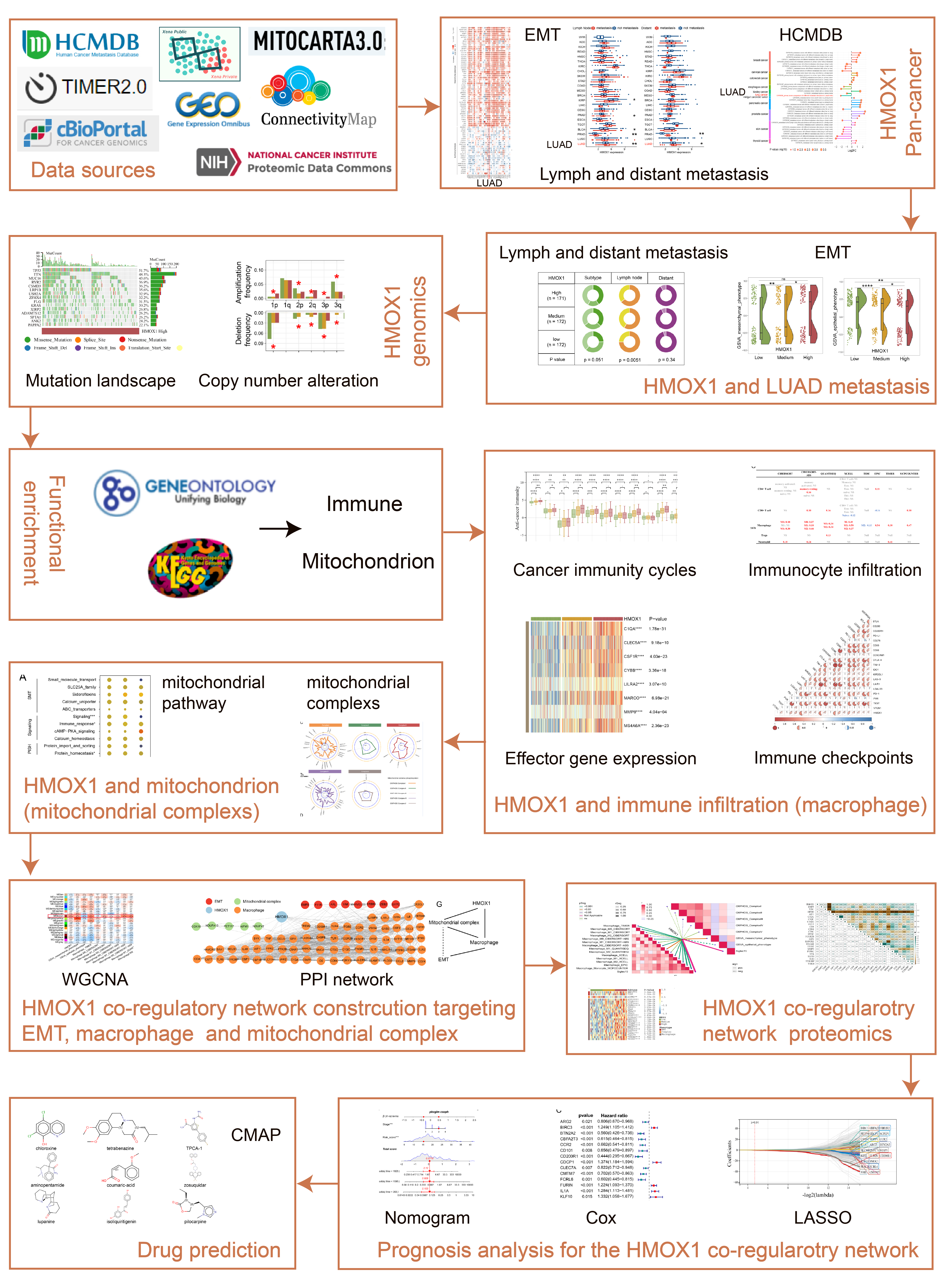

Supplement: Supplementary Figure 1 — The flow chart of data analysis. [file Image_1.tif]

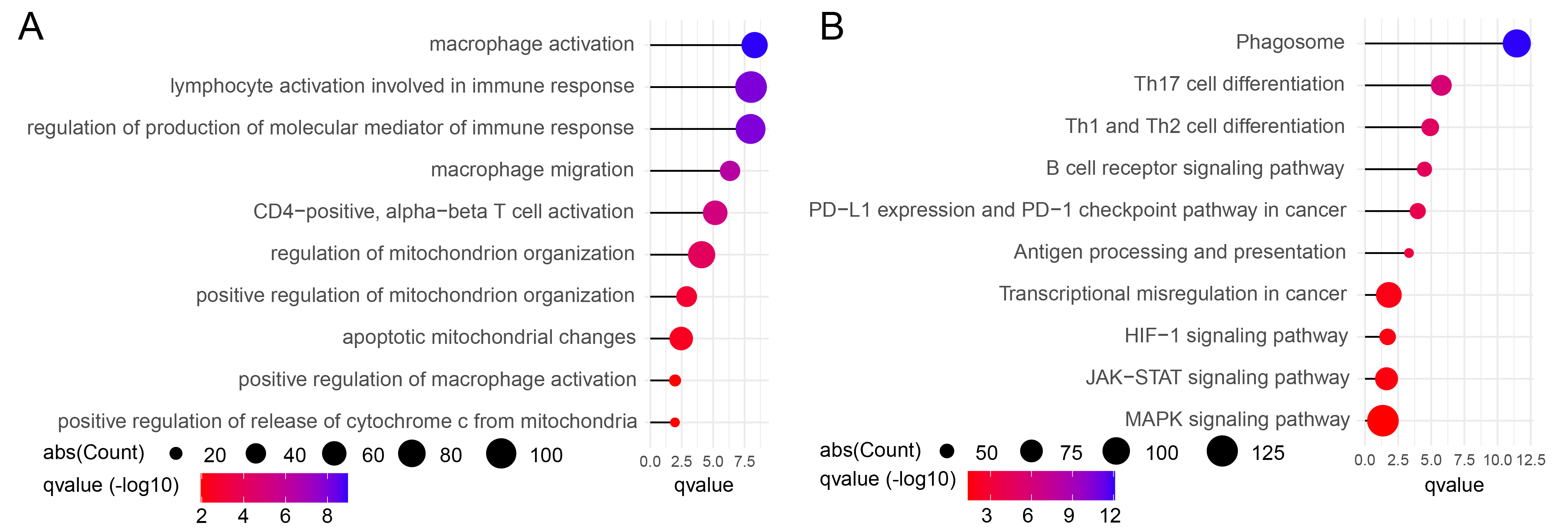

Supplement: Supplementary Figure 2 — Function enrichment between the high and low HMOX1 groups. (A) Go enrichment for the differentially expressed genes (DEGs) between the high and low HMOX1 groups. (B) Kyoto Encyclopedia of Genes and Genomes (KEGG) enrichment for the DEGs between the high and low HMOX1 groups. [file Image_2.tif]

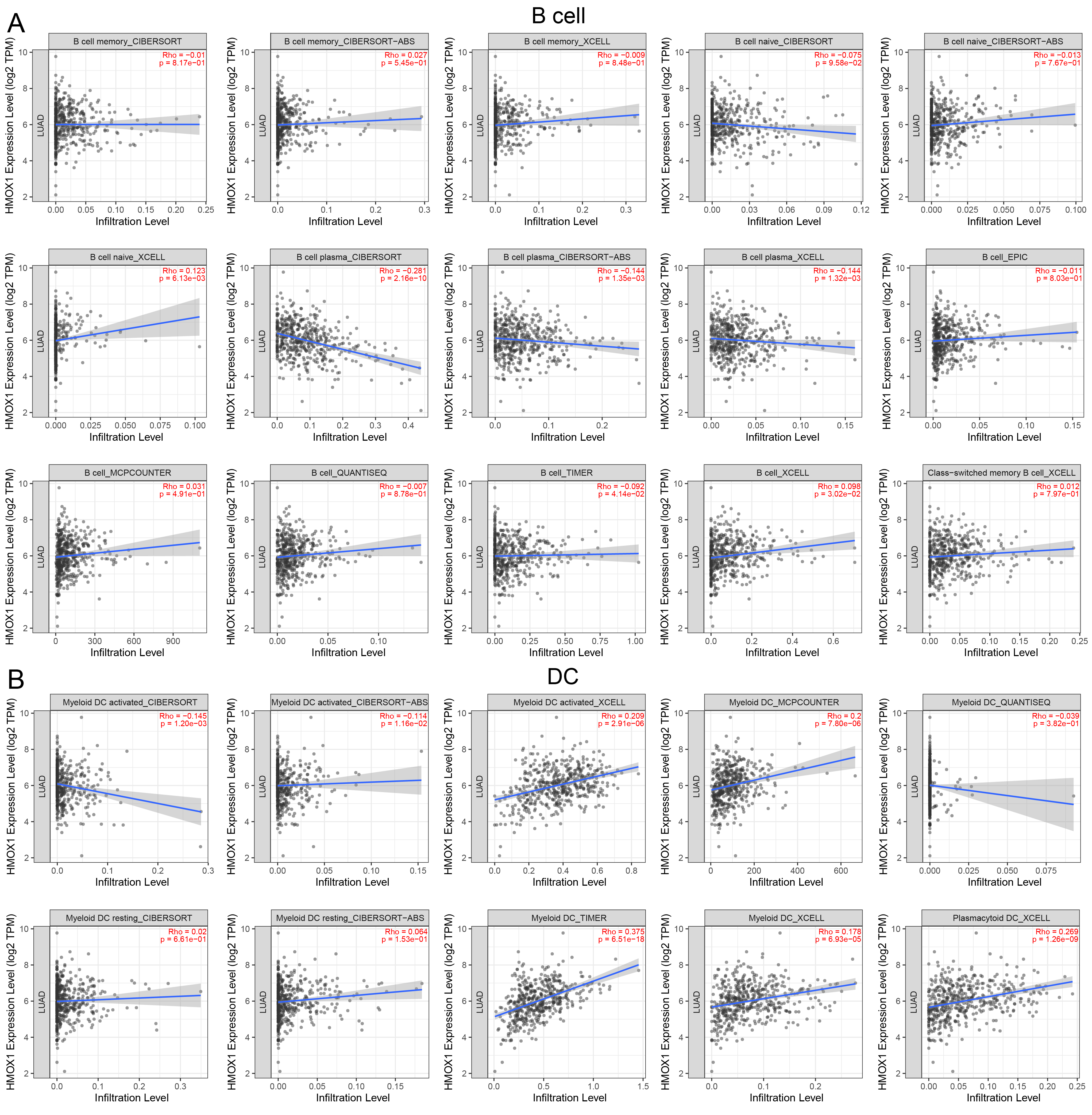

Supplement: Supplementary Figure 3 — Correlation between HMOX1 and the infiltration levels of immunocytes, including B cell (A) and dendritic cell (DC, B). [file Image_3.tif]

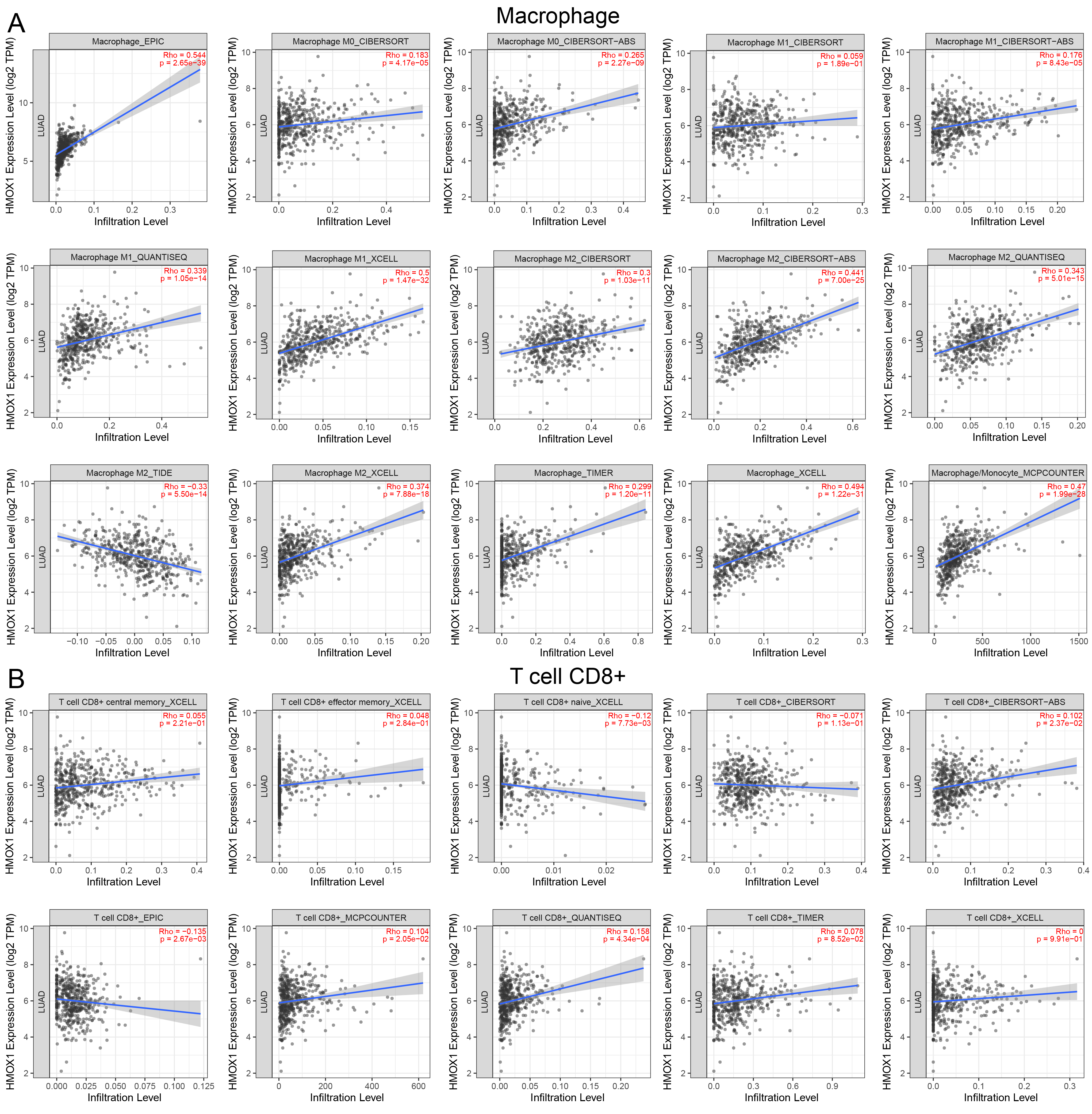

Supplement: Supplementary Figure 4 — Correlation between HMOX1 and the infiltration levels of immunocytes, including macrophage (A) and T cell CD8+ (B). [file Image_4.tif]

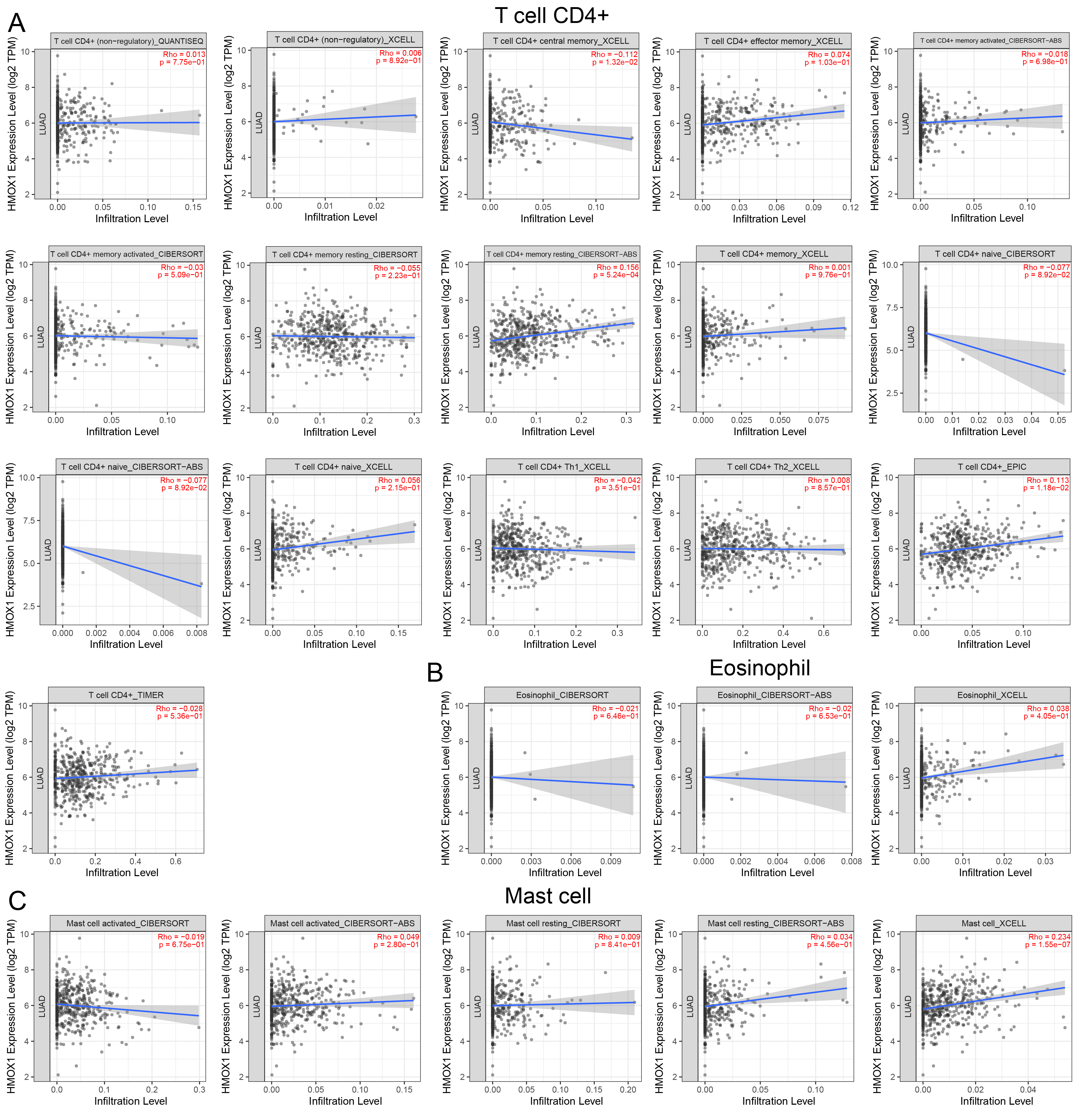

Supplement: Supplementary Figure 5 — Correlation between HMOX1 and the infiltration levels of immunocytes, including T cell CD4+ (A), Eosinphil (B). and mast cell (C). [file Image_5.tif]

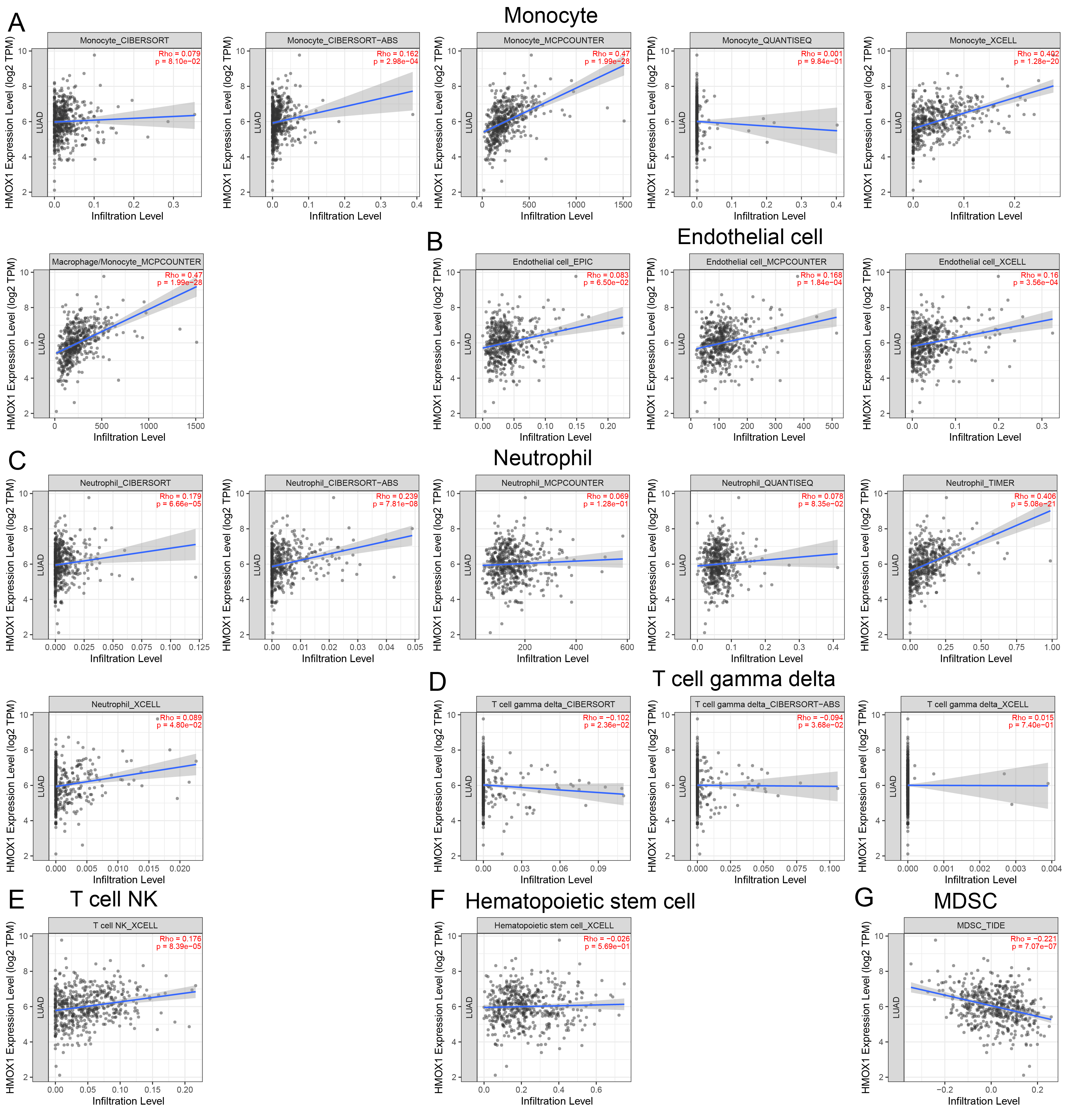

Supplement: Supplementary Figure 6 — Correlation between HMOX1 and the infiltration levels of immunocytes, including monocyte (A), endothelial cell (B), neutrophil cell (C), T cell gamma delta (D), T cell NK (E), hematopoietic stem cell (F), and myeloid-derived suppressor cell (MDCS, G). [file Image_6.tif]

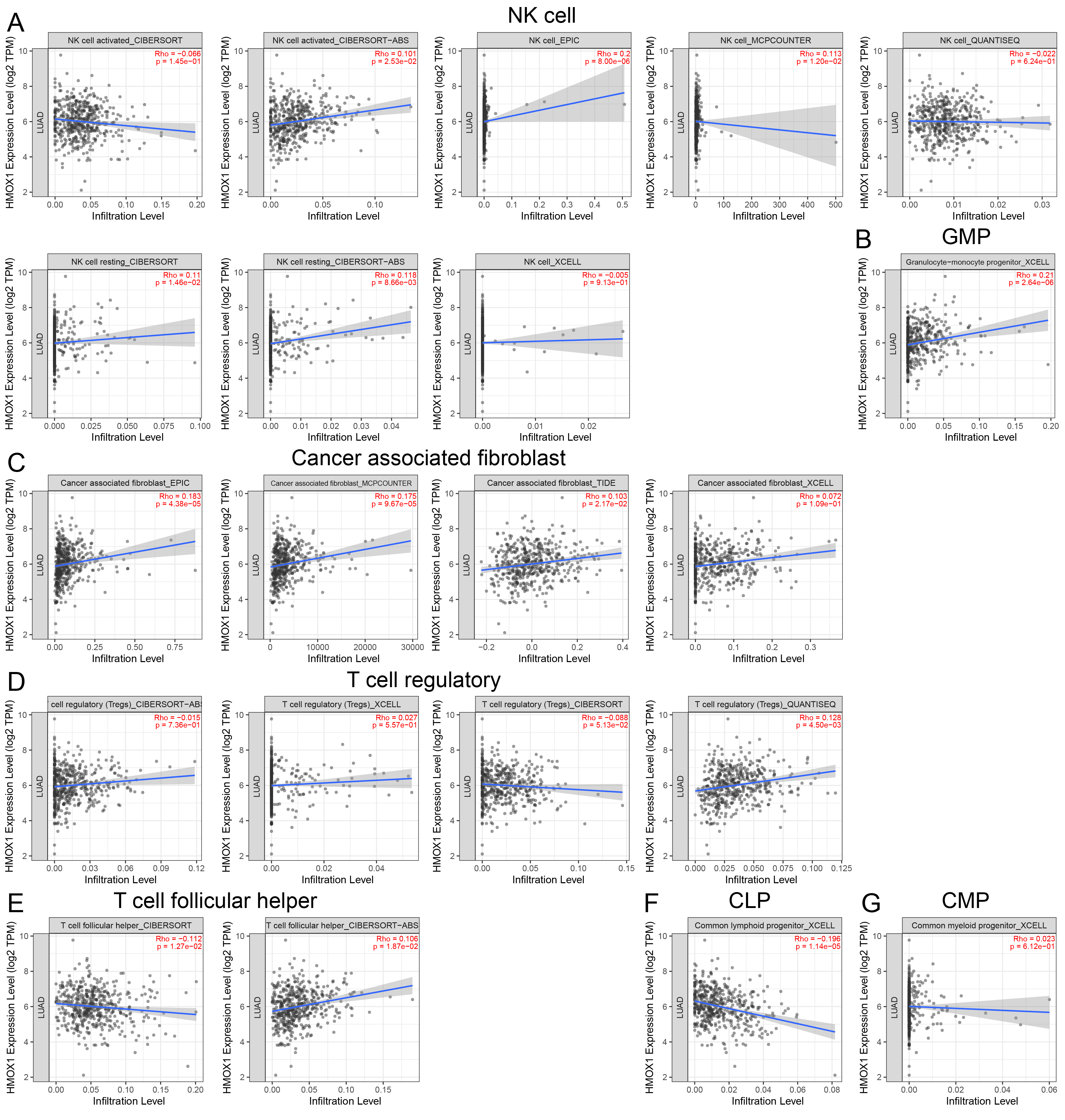

Supplement: Supplementary Figure 7 — Correlation between HMOX1 and the infiltration levels of immunocytes, including NK cell (A), common myeloid progenitor (GMP, B), cancer-associated fibroblast (C), T cell regulatory (D), T cell follicular helper (E), common lymphoid progenitor (CLP, F), and common myeloid progenitor (CMP, G). [file Image_7.tif]

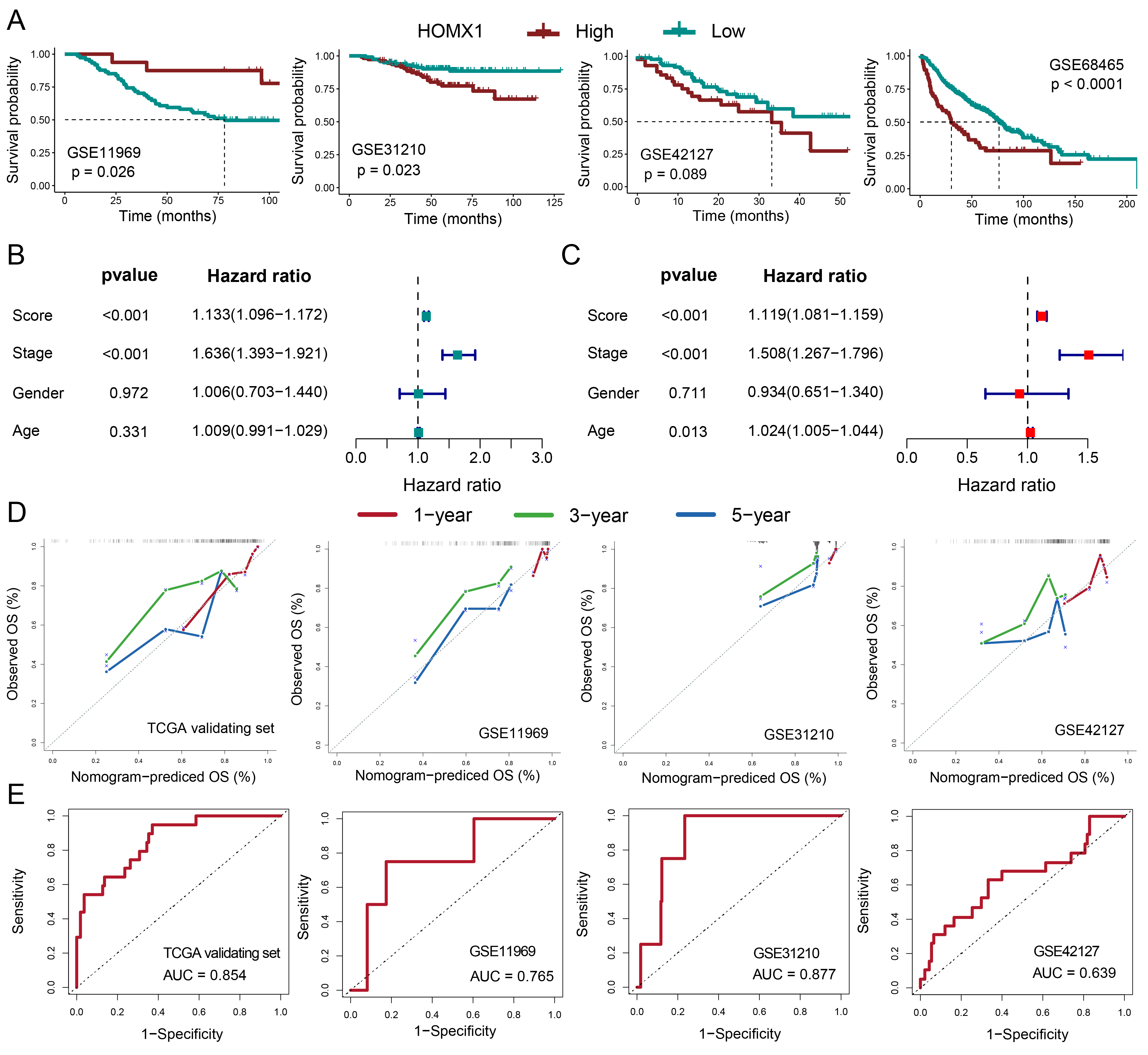

Supplement: Supplementary Figure 8 — Validation of the nomogram prognosis model in the TCGA validating and GEO cohorts. (A) Comparison of survival curves between the high and low HMOX1 groups in the GSE11969, GSE31210, GSE42127, and GSE68465 cohorts. (B) Univariate Cox analysis screened the prognosis variables. (C) Multiple Cox analysis screened the prognosis variables. (D) Calibration plots showing the correlation between actual and predicted OS rates in the TCGA validating, GSE11969, GSE31210, and GSE42127 cohorts. (E) ROC curve plot evaluating the nomogram model in the TCGA, GSE11969, GSE31210, and GSE42127 validating cohort. [file Image_8.tif]

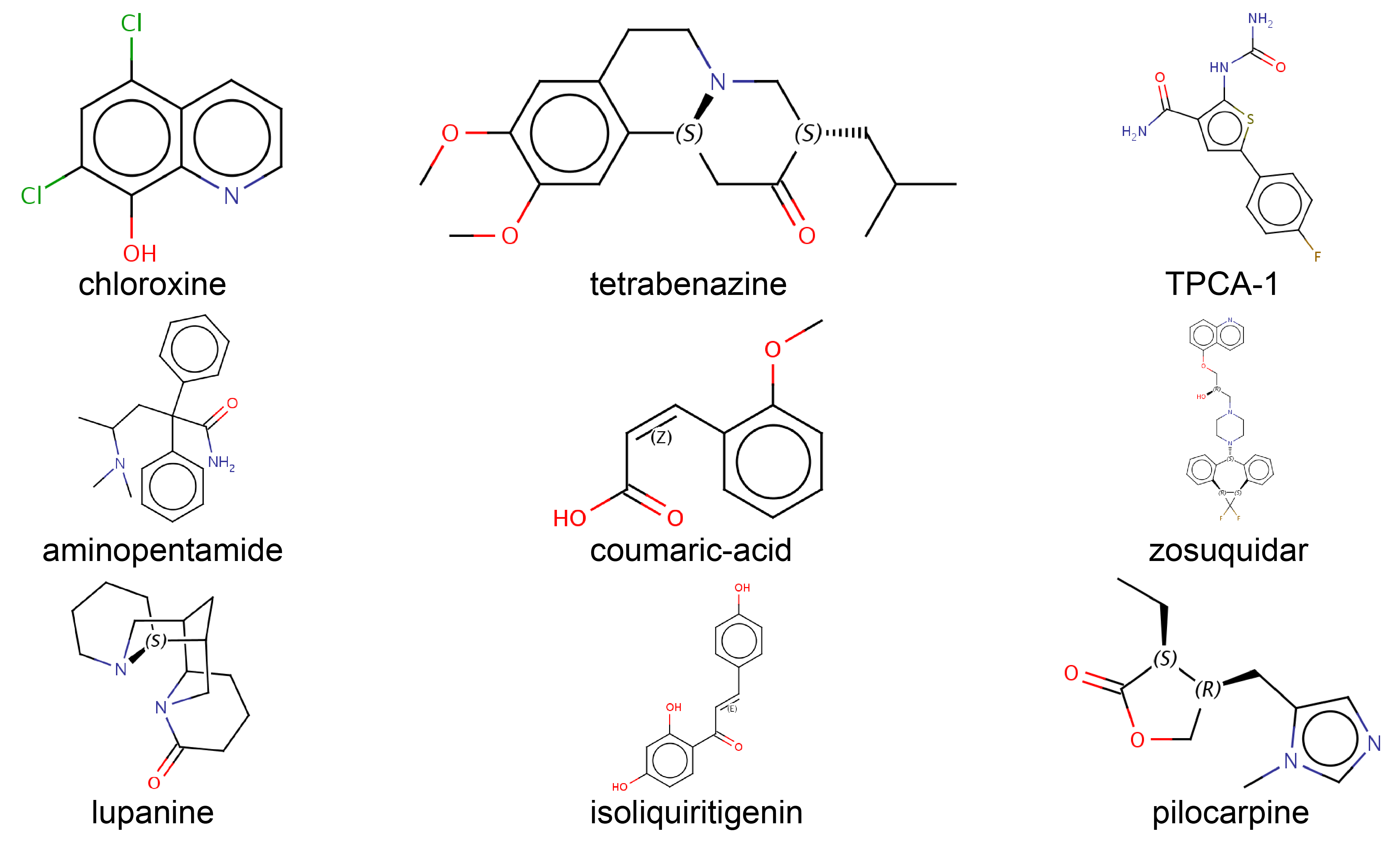

Supplement: Supplementary Figure 9 — Identifying the molecular structure of 9 small component drugs targeting the HMOX1-mediated LUAD metastasis by CMAP. [file Image_9.tif]
